# Intestinal cell type-specific communication networks underlie homeostasis and response to Western diet

**DOI:** 10.1084/jem.20221437

**Published:** 2023-03-07

**Authors:** Yu-Chen Wang, Yang Cao, Calvin Pan, Zhiqiang Zhou, Lili Yang, Aldons J. Lusis

**Affiliations:** 1 Department of Medicine, Division of Cardiology, University of California, Los Angeles, Los Angeles, CA, USA; 2 Department of Microbiology, Immunology and Molecular Genetics, University of California, Los Angeles, Los Angeles, CA, USA; 3 Eli and Edythe Broad Center of Regeneration Medicine and Stem Cell Research, University of California, Los Angeles, Los Angeles, CA, USA; 4 Jonsson Comprehensive Cancer Center, the David Geffen School of Medicine, University of California, Los Angeles, Los Angeles, CA, USA; 5 Molecular Biology Institute, University of California, Los Angeles, Los Angeles, CA, USA; 6 Department of Human Genetics, David Geffen School of Medicine at UCLA, Los Angeles, CA, USA

## Abstract

The small intestine plays a key role in immunity and mediates inflammatory responses to high fat diets. We have used single-cell RNA-sequencing (scRNA-seq) and statistical modeling to examine gaps in our understanding of the dynamic properties of intestinal cells and underlying cellular mechanisms. Our scRNA-seq and flow cytometry studies of different layers of intestinal cells revealed new cell subsets and modeled developmental trajectories of intestinal intraepithelial lymphocytes, lamina propria lymphocytes, conventional dendritic cells, and enterocytes. As compared to chow-fed mice, a high-fat high-sucrose (HFHS) “Western” diet resulted in the accumulation of specific immune cell populations and marked changes to enterocytes nutrient absorption function. Utilizing ligand–receptor analysis, we profiled high-resolution intestine interaction networks across all immune cell and epithelial structural cell types in mice fed chow or HFHS diets. These results revealed novel interactions and communication hubs among intestinal cells, and their potential roles in local as well as systemic inflammation.

## Teaser

Intercellular communications across different layers of the intestine remain poorly understood. Here, Wang et al. show the heterogeneity and developmental trajectories of intestinal intraepithelial lymphocytes, lamina propria lymphocytes, and intestinal epithelial cells, and the cell type-specific homeostatic interactomes in response to a Western diet.

## Introduction

The small intestine is the largest compartment of the immune system in mammals as well as the primary metabolic organ utilized for nutrient absorption (Chassaing et al., 2014; Mowat and Agace, 2014; Rooks and Garrett, 2016). Accumulating evidence indicates that intestinal immune and epithelial structural cells are involved in the chronic inflammation underlying obesity, atherosclerosis, inflammatory bowel disease, and many other disorders, particularly in individuals consuming high-fat high-carbohydrate “Western” diet (Arnone et al., 2021; Basson et al., 2021; Liu et al., 2021; Mukherjee et al., 2022; Nakanishi et al., 2021; Rescigno, 2014), and nearly one in two adults in the US is projected to be obese by 2030 (Ward et al., 2019). The gastrointestinal tract is secured by a series of protective layers that physically separate the circulatory system from the external milieu, including a mucus layer, epithelium layer, and lamina propria layer (Sundaram et al., 2022). Intestinal intraepithelial lymphocytes (IELs) are the major cell types that reside between the intestinal epithelial cells (IECs) forming the intestinal epithelium layer (Olivares-Villagomez and Van Kaer, 2018). IELs constitute a heterogeneous group of unique T lymphocytes and innate lymphoid cells (ILCs) that are conserved in all vertebrates (Riera Romo et al., 2016). The lamina propria contains a variety of types of myeloid cells and lamina propria lymphocytes (LPLs) and maintains close interactions with both immune cells in the epithelium layer and in the circulatory system (Cader and Kaser, 2013; Welty et al., 2013).

The functions and interactions of the intestinal intraepithelial and lamina propria immune cells, as well as IECs, remain poorly understood in mammals, especially their roles in intestinal homeostasis. Previous studies utilizing single-cell RNA-sequencing (scRNA-seq) either provided a basic characterization of the composition of intestinal cells during development (Elmentaite et al., 2021; Elmentaite et al., 2020) or investigated only the IECs (Bomidi et al., 2021; Haber et al., 2017; Wang et al., 2020). However, these studies only identified limited immune cell populations and did not spatially separate the immune cells from different layers of the intestine. Furthermore, those IEC studies were performed alone and lacked analysis of the interactions between those structural cells and immune cells. Thus, the previous studies were unable to determine the full extent of intestinal cell heterogeneity, including bona fide cell lineages, activation states, developmental trajectories, and novel cell types. To address the current knowledge gaps, we have characterized the complete cellular composition and cell type-specific signaling networks of intraepithelial immune cells, lamina propria immune cells, and epithelial structural cells derived from the small intestines of mice maintained on chow and on high-fat high-sucrose (HFHS) diet to study the intestine as a whole.

We analyzed ∼75,000 mouse immune and structural cells presented in the epithelium and lamina propria layers of the small intestine, revealing 32 previously identified cell populations and an additional 20 previously uncharacterized populations, including unique subsets of CD8αα^+^ IEL-T cells, CD8αβ^+^ IEL-T cells, CD4^+^ IEL-T cells, CD4^+^ LPL-T cells, conventional dendritic cells (cDCs), and enterocytes. We demonstrated the accumulations of distinct subsets of those populations in response to a HFHS diet and validated these by flow cytometry using independent cohorts. Utilizing the ligand–receptor analysis, we profiled the homeostatic and inflammatory circuits in the intestine, and established a signalome across intraepithelial immune cells, lamina propria immune cells, and epithelial structural cells in a cell type-specific manner. Based on these data, we identified distinct obesity-associated inflammatory and immunoregulatory pathways enriched in HFHS diet intestine. Our high-resolution, high-dimensional single-cell intestine atlas provides new insights into the functioning, development, regulatory mechanisms, and interactions of all intestinal cell types.

## Results

### Single-cell sequencing reveals a high-resolution immune landscape of intestine

**Figure 1.**
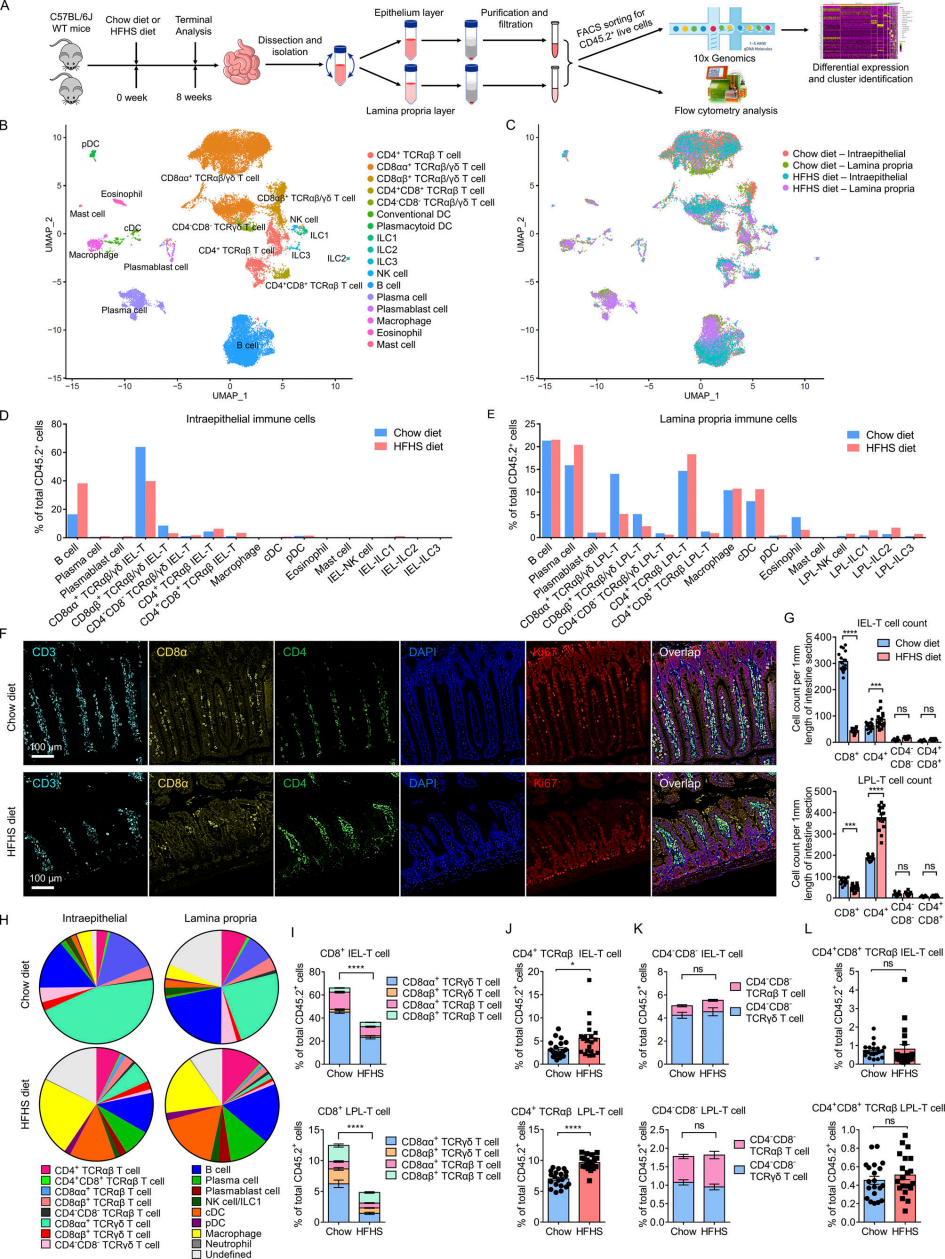
**Single-cell sequencing reveals a high-resolution immune landscape of intestine. (A)** Schematic of the experimental pipeline. C57BL/6J mice were fed on a chow diet or a HFHS diet for 8 wk and, following euthanasia, small intestines were isolated. Cells from two major intestinal compartments, the epithelium layer and lamina propria layer, were purified and then subjected to flow cytometry analysis or FACS sorting. Sorted CD45.2^+^ live cells were analyzed using droplet-based 10× Genomics Chromium scRNA-seq approach. Cells were clustered via differential gene expression for further studies. WT, wild type. **(B and C)** UMAP plot of intestine intraepithelial immune cells and lamina propria immune cells of 10 mice fed a chow diet and 10 mice fed a HFHS diet. Individual points correspond to single cells. **(B)** Annotations based on cell type analysis (Fig. S1 A). **(C)** Annotations indicating the sample original identity. IEL, intraepithelial lymphocytes; LPL, lamina propria lymphocytes. **(D and E)** Histogram showing the proportion of intraepithelial immune cells (D) and lamina propria immune cells (E) derived from 10 chow diet mice and 10 HFHS diet mice. **(F and G)** mIHC staining of small intestine ileum sections from chow diet mice and HFHS diet mice. **(F)** Sections were labeled with anti-CD3, anti-CD8α, anti-CD4, anti-Ki-67, DAPI, and images merge (100×, scale bars, 100 µm). **(G)** Histogram showing the cell counts of specific cell types of IEL-T cells and LPL-T cells per 1 mm of the intestine along the intestine length. **(H–L)** Flow cytometry analyses of major intraepithelial immune cell and lamina propria immune cell populations defined by scRNA-seq (gated as FVD^−^CD45.2^+^ cells) in an additional cohort of 16 chow diet mice and 16 HFHS diet mice (Fig. S2 D). Pie graphs indicate the proportions of major immune cell populations (H). Histograms indicate the proportions of CD8^+^ T cells (I), CD4^+^ TCRαβ T cells (J), CD4^−^CD8^−^ T cells (K), and CD4^+^CD8^+^ TCRαβ T cells (L). CD4^+^ TCRαβ T cell, CD45.2^+^TCRαβ^+^CD8α^-^CD4^+^; CD4^+^CD8^+^ TCRαβ T cell, CD45.2^+^TCRαβ^+^CD8α^+^CD4^+^; CD8αα^+^ TCRαβ T cell, CD45.2^+^TCRαβ^+^CD4^−^CD8α^+^CD8β^-^; CD8αβ^+^ TCRαβ T cell, CD45.2^+^TCRαβ^+^CD4^−^CD8α^+^CD8β^+^; CD4^−^CD8^−^ TCRαβ T cell, CD45.2^+^TCRαβ^+^CD4^−^CD8α^-^; CD8αα^+^ TCRγδ T cell, CD45.2^+^TCRγδ^+^CD4^−^CD8α^+^CD8β^-^; CD8αβ^+^ TCRγδ T cell, CD45.2^+^TCRγδ^+^CD4^−^CD8α^+^CD8β^+^; CD4^−^CD8^−^ TCRγδ T cell, CD45.2^+^TCRγδ^+^CD4^−^CD8α^-^; B cell, CD45.2^+^CD3^-^NK1.1^-^CD19^high^; plasma cell, CD45.2^+^ CD3^-^NK1.1^-^CD19^low^CD38^+^CD138^+^; Plasmablast cell, CD45.2^+^ CD3^−^NK1.1^−^CD19^low^CD38^+^CD138^−^; NK cell/ILC1, CD45.2^+^CD3^−^CD19^-^NK1.1^+^; cDC, CD45.2^+^CD11b^low^CD11c^high^I-A/I-E^high^PDCA-1^−^; pDC, CD45.2^+^CD11b^low^CD11c^high^I-A/I-E^high^PDCA-1^+^; neutrophil, CD45.2^+^CD11b^high^CD11c^−/low^Ly-6G^high^; macrophage, CD45.2^+^CD11b^high^CD11c^−/low^Ly-6G^-^F4/80^+^. FVD, fixable viability dye; ILC, innate lymphoid cell; pDC, plasmacytoid dendritic cell. Representative of 1 (A–E), 4 (F and G), and 4 (H–L) experiments. All data are presented as the mean ± SEM. ns, not significant; *P < 0.05, **P < 0.01, ***P < 0.001, and ****P < 0.0001 by two-way ANOVA (G) or by Student’s *t* test (I–L). Statistics are all two-sided. Source data are available for this figure: SourceData F1.

**Figure S1.**
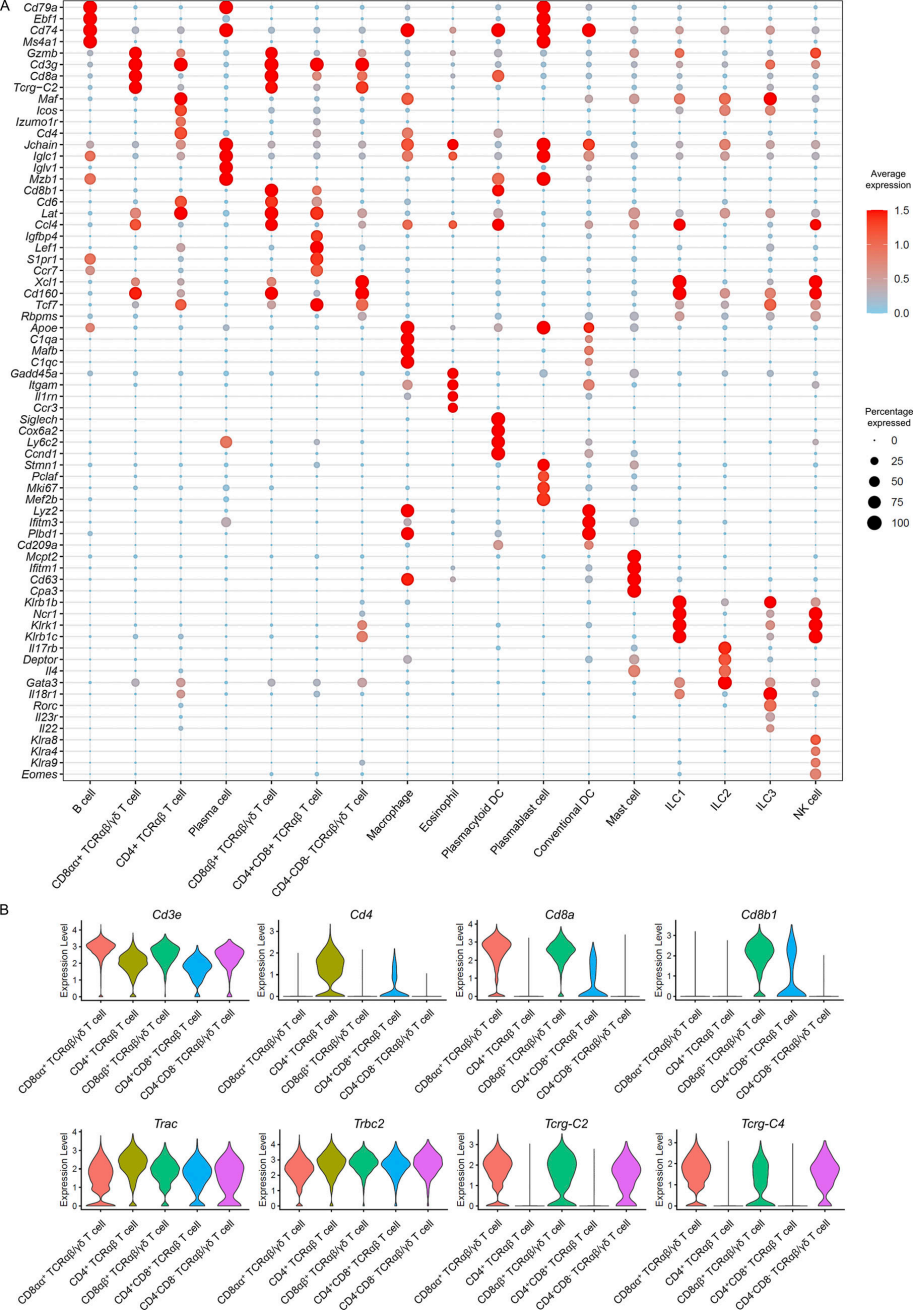
**Lineage-associated gene signatures of all CD45.2**^**+**^ **intestinal cells and intestine T cell clusters from chow diet and HFHS diet intestine. (A)** Dot plot showing the top DEGs for the populations depicted in CD45.2^+^ intestinal cells (Fig. 1 B). Color saturation indicates the strength of average gene expression, whereas the dot size reflects the percentage of each cell cluster expressing the gene. **(B)** Violin plots showing the selected genes expression among all intestine T cell clusters in CD45.2^+^ intestinal cells (Fig. 1 B).

To investigate the unbiased cellular composition of the small intestine immune system of mouse, the single-cell suspensions of immune cells of two major intestinal compartments, the epithelium layer and lamina propria layer, were isolated from 10 C57BL/6J mice fed a chow diet and 10 mice fed a HFHS diet. Cells were analyzed by flow cytometry or FACS sorted for CD45.2^+^ live cell population and then profiled utilizing droplet-based 10× Genomics Chromium scRNA-seq (Fig. 1 A). The intestine single-cell atlas obtained 30,318 quality-controlled cells. Cells were clustered based on differential expression of hallmark genes and visualized using a uniform manifold approximation and projection (UMAP) plot (Fig. 1 B and Fig. S1 A). Clustering analysis revealed 17 distinct clusters: CD8αα^+^ TCRαβ/γδ T cell, CD8αβ^+^ TCRαβ/γδ T cell, CD4^+^ TCRαβ T cell, CD4^+^CD8^+^ TCRαβ T cell, CD4^−^CD8^−^ TCRαβ/γδ T cell, cDC, plasmacytoid dendritic cell (pDC), natural killer cell (NK cell), ILC1, ILC2, ILC3, B cell, plasma cell, plasmablast cell, macrophage, eosinophil, and mast cell (Fig. 1 B). Some identified clusters contained cells from both the epithelium layer and the lamina propria layer, and they were clustered as one population in UMAP analysis although they are from completely different pools. It suggests that some IEL-T cells, like CD8αβ^+^ IEL-T cells and CD4^+^ IEL-T cells, are transcriptionally similar to their lamina propria counterparts and that they may share a common cell lineage (Fig. 1 C). The CD8αα^+^ T cell, CD8αβ^+^ T cell, and CD4^−^CD8^−^ T cell populations contained both TCRαβ T cells and TCRγδ T cells, indicating high transcriptional similarities between TCRαβ T cells and TCRγδ T cells within these clusters. Although they have different TCR gene expression, they were regarded as the same type of cells in the UMAP due to high similarities in gene expression features. However, there are clear separations between T cell clusters based on their cellular expression patterns of CD8α, CD8β, and CD4 (Fig. S1 B). Furthermore, we use the term “TCRαβ/γδ cell” to indicate scRNA-seq identified cell populations that contain both TCRαβ expressing cells and TCRγδ expressing cells. Frequency analysis of intraepithelial immune cells indicated that B cells and IEL-T cells, especially CD8αα^+^ TCRαβ/γδ IEL-T cells, are the major cell populations of intraepithelial immune cells (Fig. 1 D). Further analysis revealed dramatically decreased proportions of CD8αα^+^ TCRαβ/γδ IEL-T cells and CD8αβ^+^ TCRαβ/γδ IEL-T cells in HFHS diet intestine. In contrast, the proportions of CD4^+^ TCRαβ IEL-T cells and B cells increased (Fig. 1 D). Frequency analysis of lamina propria immune cells suggested that CD4^+^ TCRαβ LPL-T cells are the largest LPL-T cell population, and almost all myeloid cells and ILCs were localized within the lamina propria of intestine (Fig. 1 E). Further analysis indicated that the proportions of CD8αα^+^ TCRαβ/γδ LPL-T cells, CD8αβ^+^ TCRαβ/γδ LPL-T cells, and eosinophils were reduced in HFHS diet, whereas the proportions of CD4^+^ TCRαβ LPL-T cells, cDCs, and ILCs (NK cell, ILC1, ILC2 and ILC3) were increased in the HFHS diet (Fig. 1 E).

**Figure S2.**
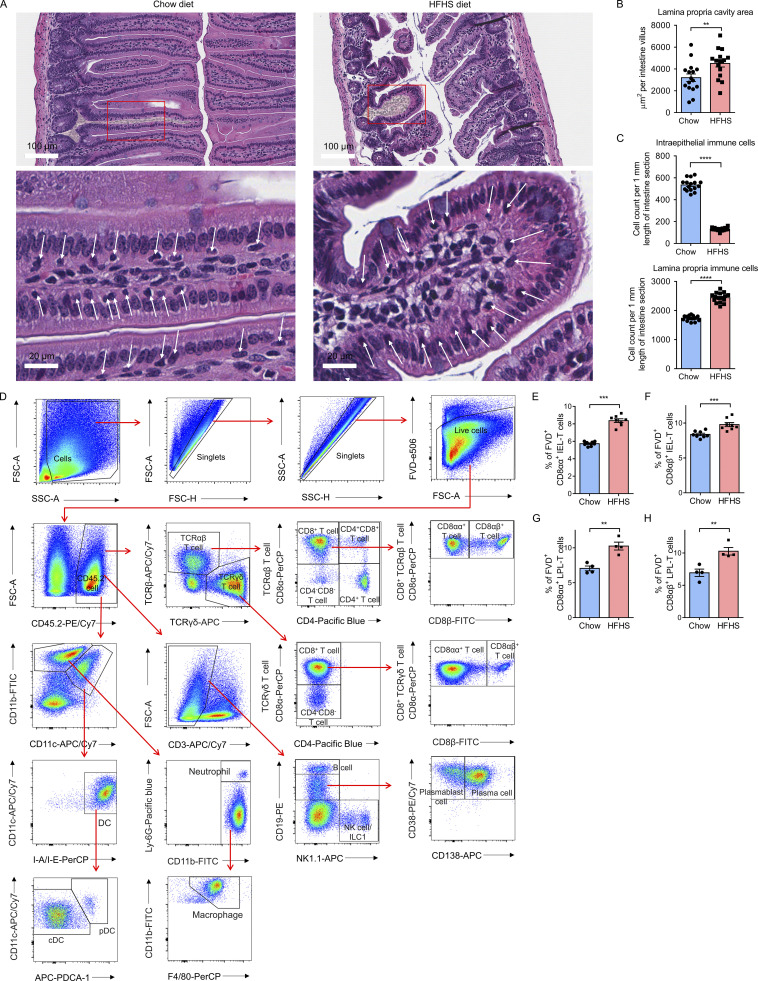
**H&E staining of small intestines and gating strategy of scRNA-seq-defined CD45.2**^**+**^ **intestinal cell populations and viability test in flow cytometry analysis. (A–C)** H&E staining of small intestine ileum sections from 16 chow diet mice and 16 HFHS diet mice. **(A)** Representative images of H&E staining analysis of chow diet and HFHS diet intestine (100×, scale bars, 100 µm, upper panel; 500×, scale bars, 20 µm, lower panel). The yellow shadows in the upper panel indicate the lamina propria area. The white arrows indicate the intraepithelial immune cells in the magnified plots. **(B)** Histogram showing area calculation of average lamina propria area per intestine villus. **(C)** Histogram showing the cell counts of intraepithelial immune cells and lamina propria immune cells per 1 mm of the intestine along the intestine length. **(D)** Representative flow cytometry analysis gating strategy of scRNA-seq-defined intraepithelial immune cell and lamina propria immune cell populations (gated as FVD^−^CD45.2^+^ cells) in the intestine (Fig. 1, H–L). CD4^+^ TCRαβ T cell, CD45.2^+^TCRαβ^+^CD8α^−^CD4^+^; CD4^+^CD8^+^ TCRαβ T cell, CD45.2^+^TCRαβ^+^CD8α^+^CD4^+^; CD8αα^+^ TCRαβ T cell, CD45.2^+^TCRαβ^+^CD4^−^CD8α^+^CD8β^−^; CD8αβ^+^ TCRαβ T cell, CD45.2^+^TCRαβ^+^CD4^−^CD8α^+^CD8β^+^; CD4^−^CD8^−^ TCRαβ T cell, CD45.2^+^TCRαβ^+^CD4^−^CD8α^−^; CD8αα^+^ TCRγδ T cell, CD45.2^+^TCRγδ^+^CD4^−^CD8α^+^CD8β^−^; CD8αβ^+^ TCRγδ T cell, CD45.2^+^TCRγδ^+^CD4^−^CD8α^+^CD8β^+^; CD4^−^CD8^−^ TCRγδ T cell, CD45.2^+^TCRγδ^+^CD4^−^CD8α^-^; B cell, CD45.2^+^CD3^−^NK1.1^−^CD19^high^; plasma cell, CD45.2^+^ CD3^−^NK1.1^−^CD19^low^CD38^+^CD138^+^; Plasmablast cell, CD45.2^+^ CD3^−^NK1.1^−^CD19^low^CD38^+^CD138^−^; NK cell/ILC1, CD45.2^+^CD3^−^CD19^−^NK1.1^+^; cDC, CD45.2^+^CD11b^low^CD11c^high^I-A/I-E^high^PDCA-1^−^; pDC, CD45.2^+^CD11b^low^CD11c^high^I-A/I-E^high^PDCA-1^+^; neutrophil, CD45.2^+^CD11b^high^CD11c^-/low^Ly-6G^high^; macrophage, CD45.2^+^CD11b^high^CD11c^−/low^Ly-6G^-^F4/80^+^. FVD, fixable viability dye; NK cell, natural killer cell; ILC, innate lymphoid cell; cDC, conventional dendritic cell; pDC, plasmacytoid dendritic cell. **(E–H)** C57BL/6J mice were fed on a chow diet or a HFHS diet for 8 wk and, following euthanasia, small intestines were isolated. Intraepithelial and lamina propria immune cells were purified and then subjected to flow cytometry analysis. To study cell viability, cells were stained with FVD followed by Fc blocking and surface marker staining. **(E and F)** Flow cytometry analyses of CD8αα^+^ IEL-T cells (E) and CD8αβ^+^ IEL-T cells (F) in chow diet and HFHS diet fed intestine (*n* = 8). **(G and H)** Flow cytometry analyses of CD8αα^+^ LPL-T cells (G) and CD8αβ^+^ LPL-T cells (H) in chow diet and HFHS diet fed intestine (*n* = 4). CD8αα^+^ T cells, CD45.2^+^CD3^+^CD4^−^CD8α^+^CD8β^−^ cells; CD8αβ^+^ T cells, CD45.2^+^CD3^+^CD4^−^CD8α^+^CD8β^+^ cells. FVD, fixable viability dye. Representative of 5 (A–C) and 4 (D–H) experiments. All data are presented as the mean ± SEM. *P < 0.05, **P < 0.01, ***P < 0.001, and ****P < 0.0001 by Student’s *t* test (B, C, and E–H). Statistics are all two-sided. Source data are available for this figure: SourceData FS2.

We next used an extensive number of mice in pathology examinations and flow cytometry to verify every observation regarding alternations in cell type proportion from scRNA-seq analysis. Hematoxylin and eosin (H&E) staining was conducted to investigate the morphological change and absolute quantification of immune cells. In response to HFHS diet, intestinal villi exhibited dysmorphology and lamina propria cavities became enlarged (Fig. S2, A and B). The intraepithelial immune cells could be distinguished by their location—between IECs and outside of the lamina propria cavity. The diet resulted in a decrease in intraepithelial immune cells and an increase in infiltration of lamina propria immune cells (Fig. S2, A and C). Multiplex-immunohistochemistry (mIHC) examination showed that IEL-T cells were dominantly CD8^+^ IEL-T cells (CD8αα^+^ IEL-T cells and CD8αβ^+^ IEL-T cells), and the majority of LPL-T cells were CD4^+^ LPL-T cells (Fig. 1 F). In accordance with our scRNA-seq analysis, CD8^+^ IEL-T cells and CD8^+^ LPL-T cells decreased, whereas CD4^+^ LPL-T cells and CD4^+^ IEL-T cells increased in HFHS diet intestine (Fig. 1 G).

To further interrogate the changes and dissect the population of TCRαβ T cells and TCRγδ T cells, an independent cohort of 20 chow diet-fed mice and 20 HFHS diet-fed mice was analyzed by flow cytometry for major immune cell populations (Fig. 1 H and Fig. S2 D). The undefined cell populations here might include monocytes, basophils, mast cells, ILC2s, ILC3s, and pre-cDCs (Sun et al., 2015; Varol et al., 2010). The decreased CD8^+^ IEL-T cells in the HFHS diet intestine resulted primarily from decreases in the CD8αα^+^ TCRγδ T cells, CD8αβ^+^ TCRγδ T cells and CD8αα^+^ TCRαβ T cells, whereas the decreased CD8^+^ LPL-T cells resulted primarily from the reduction of CD8αα^+^ TCRγδ T cells, CD8αβ^+^ TCRγδ T cells, and CD8αβ^+^ TCRαβ T cells (Fig. 1 I). Flow cytometry further supported our scRNA-seq analysis, demonstrating the increase of CD4^+^ IEL-T cells and CD4^+^ LPL-T cells in HFHS diet intestine (Fig. 1 J). No significant changes were observed in CD4^−^CD8^−^ TCRαβ/γδ T cells and CD4^+^CD8^+^ TCRαβ T cells in both the epithelium layer and lamina propria (Fig. 1, K and L).

To determine if the decrease of CD8^+^ T cells upon feeding the HFHS diet is due to cell death, we used fixable viability dye (FVD) to measure the viability of CD8^+^ IEL-T cells and CD8^+^ LPL-T cells in the intestine upon HFHS diet feeding and chow diet feeding. The results show that the viability of CD8αα^+^ IEL-T and CD8αβ^+^ IEL-T cells significantly decreased after HFHS diet feeding (Fig. S2, E and F). Furthermore, the viability of CD8αα^+^ LPL-T and CD8αβ^+^ LPL-T cells also decreased after HFHS diet feeding (Fig. S2, G and H). These results indicate that the HFHS diet does induce cell death in the intraepithelial immune cells, including both CD8αα^+^ T cells and CD8αβ^+^ T cells in the intestine.

These results indicated that a HFHS diet is associated with dramatic changes in intestine CD8^+^ T cells, CD4^+^ T cells, and cDCs in both the epithelium layer and the lamina propria. These cells likely contain heterogeneous populations or new cell subsets that have not previously been characterized, suggesting the necessity of further subclustering.

### Unique CD8^+^ IEL-T cell subsets accumulate in HFHS diet intestine

**Figure 2.**
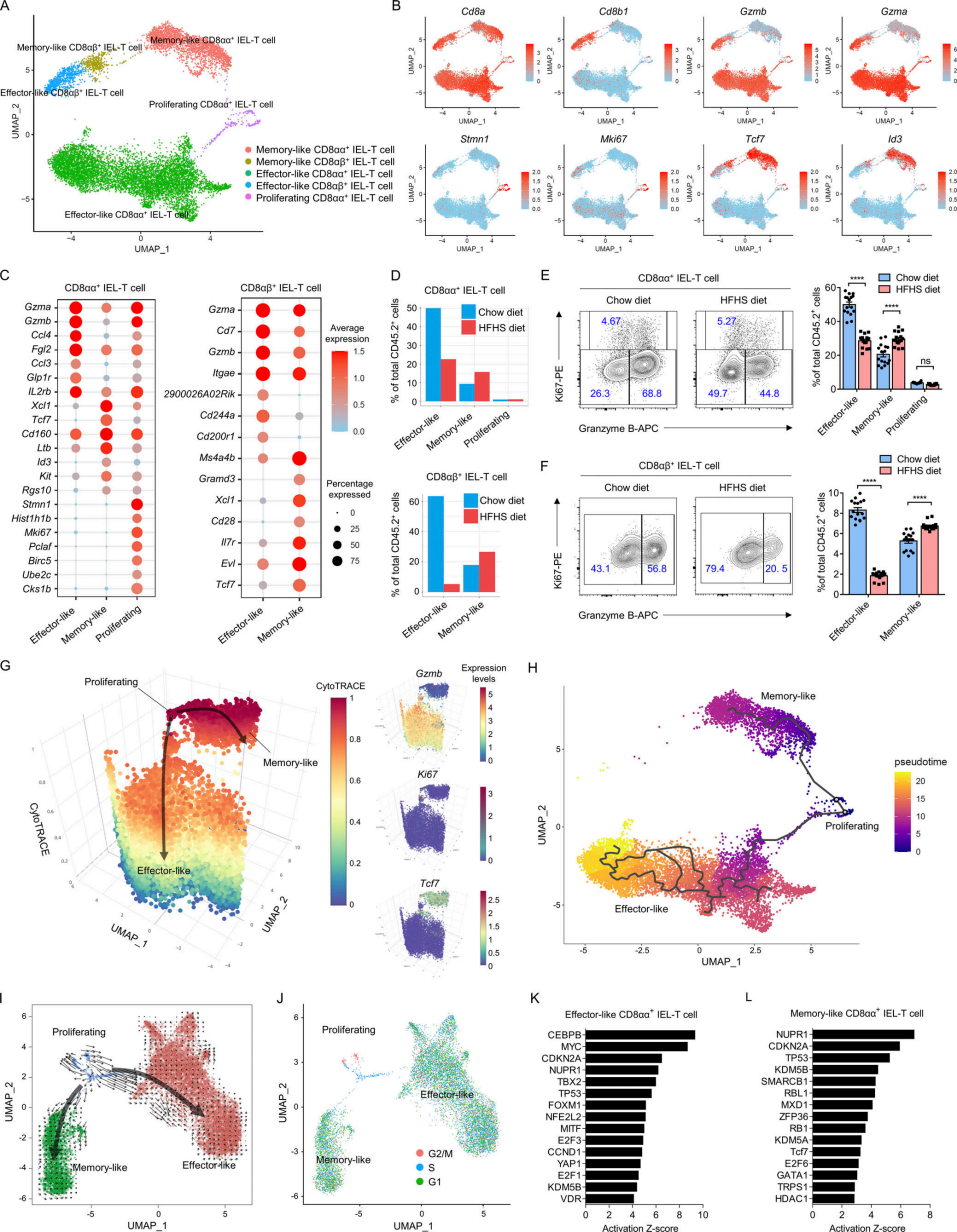
**Unique CD8**^**+**^ **IEL-T cell subsets accumulate in HFHS diet intestine. (A and B)** UMAP plot of mouse intestine CD8^+^ IEL-T cells derived from a cohort of 10 chow diet mice and 10 HFHS diet mice. Individual points correspond to single cells. **(A)** Cluster analysis yields five distinct clusters comprising CD8αα^+^ IEL-T cells and CD8αβ^+^ IEL-T cells. **(B)** Feature plots showing the selected signature genes projection on UMAP. Intensity of gene expression in each cell was indicated in color saturation. **(C)** Dot plot showing selected top DEGs for the populations depicted in CD8αα^+^ IEL-T cells and CD8αβ^+^ IEL-T cells. Color saturation indicates the strength of average gene expression, whereas the dot size reflects the percentage of each cell cluster expressing the gene. **(D)** Histogram showing the proportions of CD8αα^+^ IEL-T cells and CD8αβ^+^ IEL-T cells derived from 10 chow diet mice and 10 HFHS diet mice. **(E and F)** Flow cytometry analyses of scRNA-seq-defined CD8αα^+^ IEL-T cell (gated as CD45.2^+^CD3^+^CD4^−^CD8α^+^CD8β^−^ cells; E) and CD8αβ^+^ IEL-T cell (gated as CD45.2^+^CD3^+^CD4^−^CD8α^+^CD8β^+^ cells; F) populations in an additional cohort of 16 chow diet mice and 16 HFHS diet mice (Fig. S3 A). Effector-like CD8αα^+^ IEL-T cells, Granzyme B^+^Ki67^−^; memory-like CD8αα^+^ IEL-T cells, Granzyme B^−^Ki67^−^; proliferating CD8αα^+^ IEL-T cells, Ki67^+^; effector-like CD8αβ^+^ IEL-T cells, Granzyme B^+^; memory-like CD8αβ^+^ IEL-T cells, Granzyme B^−^. **(G)** Visualizing the predicted differentiation using a CytoTRACE 3D plot of CD8αα^+^ IEL-T cells showing the CytoTRACE score component with UMAP coordinates. The color indicates the CytoTRACE score from 1 (red, lowest levels of differentiation) to 0 (blue, highest levels of differentiation). Feature plots showing the selected signature genes projection on 3D plot. Intensity of gene expression in each cell was indicated in color saturation from low (red) to high (blue). **(H)** Monocle analysis of the CD8αα^+^ IEL-T cells. The color indicates pseudotime directionality projection on UMAP from the earliest (blue) to the latest (yellow). **(I)** RNA-velocity analysis of CD8αα^+^ IEL-T cell subclusters with velocity field projected onto the UMAP plot. Arrows show the local average velocity evaluated on a regular grid and indicate the extrapolated future states of cells. **(J)** Cell cycle analysis of the CD8αα^+^ IEL-T cells. Predicted classification of each cell in either G2/M (red), S (blue), or G1 (green) phase was projected on UMAP. **(K and L)** IPA analysis of upstream transcriptional regulators of the transition from proliferating CD8αα^+^ IEL-T cells to either effector-like CD8αα^+^ IEL-T cells (K) or memory-like CD8αα^+^ IEL-T cells based on DEGs (L). Representative of 1 (A–D and G–L), and 4 (E and F) experiments. All data are presented as the mean ± SEM. ns, not significant; *P < 0.05, **P < 0.01, ***P < 0.001, and ****P < 0.0001 by two-way ANOVA (E and F). Statistics are all two-sided.

**Figure S3.**
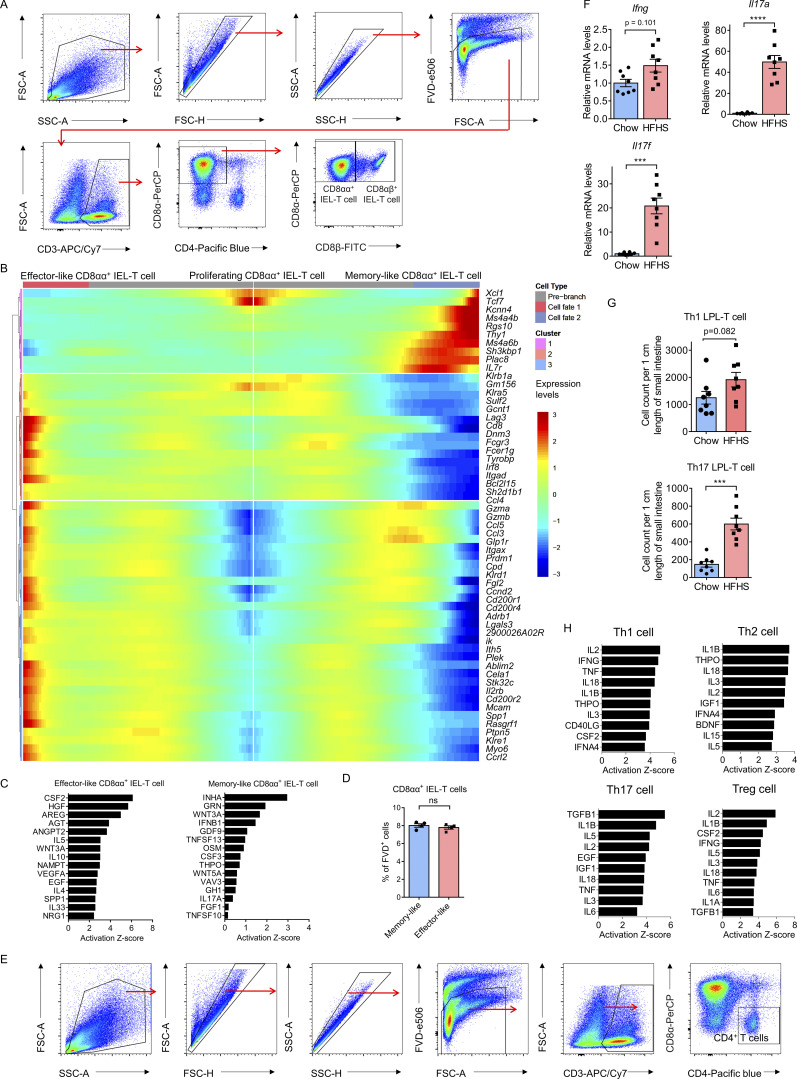
**Unique T cell subsets accumulate in HFHS diet intestine. (A)** Representative flow cytometry analysis gating strategy of scRNA-seq-defined CD8αα^+^ IEL-T cells (gated as FVD^−^CD45.2^+^CD3^+^CD4^−^CD8α^+^CD8β^−^ cells) and CD8αβ^+^ IEL-T cells (gated as FVD^−^CD45.2^+^CD3^+^CD4^−^CD8α^+^CD8β^+^ cells) for intracellular staining of cytotoxicity molecules and transcription factors (Fig. 2, E and F). FVD, fixable viability dye. **(B)** Bifurcation heatmap of enriched genes for effector-like CD8αα^+^ IEL-T cell (left), proliferating CD8αα^+^ IEL-T cell (middle) and memory-like CD8αα^+^ IEL-T cell (right). Color indicates increased (red) or decreased (blue) gene expression. **(C)** IPA of secreted upstream regulators of the transition from proliferating CD8αα^+^ IEL-T cells to either effector-like CD8αα^+^ IEL-T cells or memory-like CD8αα^+^ IEL-T cells. **(D)** Histogram of flow cytometry analyses of scRNA-seq-defined effector-like and memory-like CD8αα^+^ IEL-T cells in mice fed with a HFHS diet (*n* = 4). C57BL/6J mice were fed on a chow diet or a HFHS diet for 8 wk and, following euthanasia, small intestines were isolated. Intraepithelial immune cells were purified and then subjected to flow cytometry analysis. To study cell viability, cells were stained with FVD followed by Fc blocking and surface marker staining. CD8αα^+^ IEL-T cells, CD45.2^+^CD3^+^CD4^−^CD8α^+^CD8β^−^ cells; effector-like CD8αα^+^ IEL-T cells, Granzyme B^+^Ki67^-^; memory-like CD8αα^+^ IEL-T cells, Granzyme B-Ki67^−^; FVD, fixable viability dye. **(E)** Representative flow cytometry analysis gating strategy of scRNA-seq-defined CD4^+^ IEL-T cells and CD4^+^ LPL-T cells (gated as FVD^−^CD45.2^+^CD3^+^CD8α^−^CD4^+^ cells) for intracellular cytokine staining (Fig. 3, E and F). FVD, fixable viability dye. **(F)** Whole intestinal tissue from ileum to jejunum were collected and analyzed using RT-QPCR. Histogram showing the mRNA expression levels of *Il6*, *Il17a*, and *Il17f*. *N* = 8. **(G)** Flow cytometry analyses of the absolute cell count of Th17 LPL-T cells (gated as CD45.2^+^CD3^+^CD8α^−^CD4^+^IL17A^+^) from lamina propria immune cells. *n* = 8. **(H)** IPA of putative secreted upstream regulators of the transition from memory-like CD4^+^ T cells to Th1, Th2, Th17, or Treg cells. Representative of 2 (D, F, and G) and 4 (A and E) experiments. All data are presented as the mean ± SEM. ns, not significant; *P < 0.05, **P < 0.01, ***P < 0.001, and ****P < 0.0001 by Student’s *t* test (D). Statistics are all two-sided.

Previous studies from mice and humans suggest that CD8αα^+^ IEL-T cells, including CD8αα^+^ TCRγδ IEL-T cells and CD8αα^+^ TCRαβ IEL-T cells, are all natural IELs that possess the potential for self-renewal within the intestine epithelium layer (McDonald et al., 2018; Ruscher et al., 2017), whereas CD8αβ^+^ IEL-T cells include conventional CD8αβ^+^ TCRαβ T cells and a novel CD8αβ^+^ TCRγδ T cell population (Cheroutre et al., 2011; Kadivar et al., 2016; Olivares-Villagomez and Van Kaer, 2018). To further examine whether cellular heterogeneity existed in CD8^+^ IEL-T cells, we assessed a total of 11,677 pooled cells from all CD8^+^ IEL-T cells of 10 chow diet mice and 10 HFHS diet mice and UMAP visualization revealed five distinct clusters (Fig. 2 A). Besides the identification of CD8αα^+^ IEL-T cells and CD8αβ^+^ IEL-T cells based on the distribution of *Cd8a* and *Cd8b1* gene expression, we found that each of them contained a population expressing high levels of cytotoxicity genes such as *Gzma* and *Gzmb* and a separate population expresses high levels of memory T cell signature genes such as *Tcf7* and *Id3*. In addition, a third population was observed in CD8αα^+^ IEL-T cells which expressed high levels of stem cell-related genes such as *Stmn1* and *Mki67* (Fig. 2 B). Based on the top variable expressed genes in each subcluster, our analysis identified three populations of CD8αα^+^ IEL-T cells (effector-like CD8αα^+^ IEL-T cells, memory-like CD8αα^+^ IEL-T cells, and proliferating CD8αα^+^ IEL-T cells) and two populations of CD8αβ^+^ IEL-T cells (effector-like CD8αβ^+^ IEL-T cells and memory-like CD8αβ^+^ IEL-T cells; Fig. 2 C). Assessment of the frequency of each cell cluster indicated that effector-like IEL-T cells are the major subpopulation of both CD8αα^+^ IEL-T cells and CD8αβ^+^ IEL-T cells, and revealed a shift in frequency from effector-like IEL-T cells in chow diet intestines to memory-like IEL-T cell in HFHS diet intestines (Fig. 2 D). To further confirm the presence of scRNA-seq-identified cell lineages of CD8αα^+^ IEL-T cells, we harvested intraepithelial immune cells from an additional cohort of mice (16 chow diet, 16 HFHS diet). Flow cytometry experiments corroborated the results from our scRNA-seq clustering, identifying three distinct CD8αα^+^ IEL-T cell populations and two CD8αβ^+^ IEL-T cell populations based on endogenous Ki67 and Granzyme B expression (Fig. 2, E and F; and Fig. S3 A). Furthermore, in support of our scRNA-seq results, they demonstrated that CD8αα^+^ IEL-T cells and CD8αβ^+^ IEL-T cells decreased their effector-like cell proportions and increased memory-like cell proportions in HFHS diet intestine (Fig. 2, E and F).

UMAP visualization of CD8αα^+^ IEL-T cells showed that the identified proliferating CD8αα^+^ IEL-T cells are transcriptionally close to effector-like CD8αα^+^ IEL-T cells and memory-like CD8αα^+^ IEL-T cells, suggesting that proliferating CD8αα^+^ IEL-T cells represent a committed precursor to both populations (Fig. 2 A). To test this hypothesis, we utilized CytoTRACE analysis, a trajectory reconstruction analysis using gene counts and expression to predict differentiation states in scRNA-seq datasets (Gulati et al., 2020). The 3-dimension (3D) plot suggested that proliferating CD8αα^+^ IEL-T cells represented the least-differentiated cells and that progressions toward either the effector-like CD8αα^+^ IEL-T cell or memory-like CD8αα^+^ IEL-T cell status increased the levels of differentiation (Fig. 2 G). We also utilized Monocle to perform single-cell trajectory analysis of how cells choose between one of several possible end states, placing them along a trajectory corresponding to a biological process (Trapnell et al., 2014). Monocle pseudotime analysis further supported the bifurcating developmental trajectories from proliferating CD8αα^+^ IEL-T cells to memory-like CD8αα^+^ IEL-T cell and effector-like CD8αα^+^ IEL-T cell fates (Fig. 2 H). Furthermore, RNA-velocity analysis was utilized to determine the transcriptional fates of the proliferating CD8αα^+^ IEL-T cell population. RNA velocity uses the time derivative of the gene expression state, which can be directly estimated by distinguishing between unspliced and spliced mRNAs in common scRNA-seq datasets to predict the future state of individual cells on a timescale (La Manno et al., 2018). Projection of the velocity field arrows onto the UMAP plot extrapolated future states of proliferating CD8αα^+^ IEL-T cells to both effector-like CD8αα^+^ IEL-T cell and memory-like CD8αα^+^ IEL-T cell populations (Fig. 2 I). Cell cycle analysis (Nestorowa et al., 2016) predicted classification of each cell in either the G2/M, S, or G1 phase. Proliferating CD8αα^+^ IEL-T cells exhibited a highly proliferative status with all cells in either G2/M or S phase, whereas effector-like CD8αα^+^ IEL-T cells and memory-like CD8αα^+^ IEL-T cells were mainly in the G1 phase, consistent with the predicted transition (Fig. 2 J). Manually averaged principal curves assigned on CytoTRACE analysis and RNA-velocity, and branch analysis on Monocle trajectory suggest clear bifurcating developmental trajectories from proliferating CD8αα^+^ IEL-T cells to effector-like CD8αα^+^ IEL-T cells and memory-like CD8αα^+^ IEL-T cells (Fig. 2, G–I). Analysis of differentially expressed genes (DEGs) between the effector-like CD8αα^+^ IEL-T cell and memory-like CD8αα^+^ IEL-T cell fates from proliferating CD8αα^+^ IEL-T cells showed a clear bifurcation in gene expression patterns (Fig. S3 B). Ingenuity pathway analysis (IPA) implicated dramatic differences in upstream signaling regulators between the transition to the effector-like CD8αα^+^ IEL-T cell fate and the transition to the memory-like CD8αα^+^ IEL-T cell fate, further suggesting a developmental switch to the memory-like CD8αα^+^ IEL-T cell fate in response to the HFHS diet (Fig. 2, K and L; and Fig. S3 C). To investigate whether CD8αα^+^ memory-like IEL-T cells and CD8αα^+^ effector-like IEL-T cells differ in sensitivity to cell death by the HFHS diet, we measured their viability through intracellular staining. The result indicated that there are no significant differences in cell viability between CD8αα^+^ memory-like IEL-T cells and CD8αα^+^ effector-like IEL-T cells (Fig. S3 D). This result indicates that development rather than sensitivity might be the major reason for the shift in frequency.

These results suggest a previously unidentified shared precursor exists in the CD8αα^+^ IEL-T cells, and that the accumulation of memory-like CD8αα^+^ IEL-T cells during HFHS feeding may be due to increased differentiation from proliferating CD8αα^+^ IEL-T cells.

### Distinct CD4^+^ T cell subsets accumulate in HFHS diet intestine

**Figure 3.**
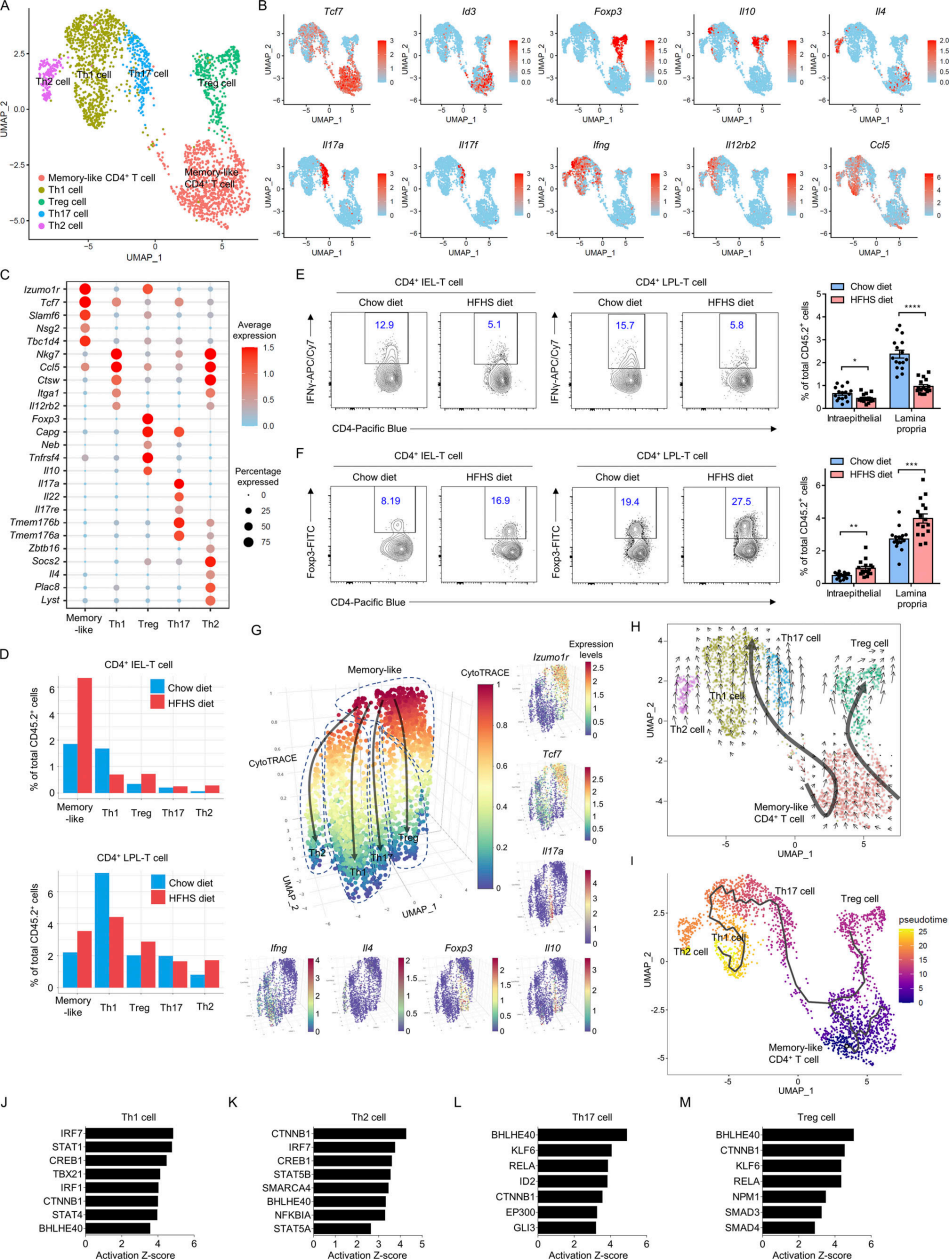
**Distinct CD4**^**+**^ **T cell subsets accumulate in HFHS diet intestine. (A and B)** UMAP plot of mouse intestine CD4^+^ T cells derived from a cohort of 10 chow diet mice and 10 HFHS diet mice, where individual points correspond to single cells. **(A)** Cluster analysis yields five distinct clusters from CD4^+^ IEL-T cells and CD4^+^ LPL-T cells. **(B)** Feature plots showing the selected signature genes projection on UMAP. Intensity of gene expression in each cell was indicated in color saturation. Th, T helper; Treg cell, regulatory T cell. **(C)** Dot plot showing selected top DEGs for the populations depicted in CD4^+^ T cells. Color saturation indicates the strength of average gene expression, whereas the dot size reflects the percentage of each cell cluster expressing the gene. **(D)** Histogram showing the proportions of CD4^+^ IEL-T cells and CD4^+^ LPL-T cells derived from 10 chow diet mice and 10 HFHS diet mice. **(E and F)** Flow cytometry analyses of scRNA-seq-defined Treg cells (E) and Th1 cells (F) in CD4^+^ IEL-T cell and CD4^+^ LPL-T cell populations (gated as CD45.2^+^CD3^+^CD8α^−^CD4^+^ cells) in an additional cohort of 16 chow diet mice and 16 HFHS diet mice (Fig. S3 E). Treg cells, Foxp3^+^; Th1 cells, IFNγ^+^. **(G)** Visualizing the predicted differentiation using a CytoTRACE 3D plot of CD4^+^ T cells showing the CytoTRACE score component with UMAP coordinates. The color indicates the levels of CytoTRACE score from 1 (red, lowest levels of differentiation) to 0 (blue, highest levels of differentiation). Feature plots showing the selected signature genes projection on 3D plot. Intensity of gene expression in each cell was indicated in color saturation from low (red) to high (blue). **(H)** RNA-velocity analysis of CD4^+^ T cell subclusters with velocity field projected onto the UMAP plot. Arrows show the local average velocity evaluated on a regular grid and indicate the extrapolated future states of cells. **(I)** Monocle analysis of the CD4^+^ T cells. The color indicates pseudotime directionality projection on UMAP from the earliest (blue) to the latest (yellow). **(J–M)** IPA analysis of upstream transcriptional regulators of the transition from memory-like CD4^+^ T cells to Th1 cells (J), Th2 cells (K), Th17 cells (L), or Treg cells (M) based on DEGs. Representative of 1 (A–D and G–M), and 4 (E and F) experiments. All data are presented as the mean ± SEM. *P < 0.05, **P < 0.01, ***P < 0.001, and ****P < 0.0001 by Student’s *t* test (E and F). Statistics are all two-sided.

Previous studies from mice and humans indicate that both CD4^+^ TCRαβ IEL-T cells and CD4^+^ TCRαβ LPL-T cells are conventional T cell populations, and characterization of their subsets has not previously been described. To examine whether further cellular heterogeneity existed, we pooled all CD4^+^ IEL-T cells and CD4^+^ LPL-T cells from the intestines of 10 HFHS diet mice and 10 chow diet mice and UMAP visualization revealed five distinct populations (Fig. 3 A). These included four classic CD4^+^ T cell populations: T helper 1 (Th1) cells by expression of *Ifng*,* Il12rb2*, and *Ccl5*; Th2 cells by expression of *Il4;* Th17 cells by expression of *Il22*, *Il17a*, and *Il17f*; and regulatory T cells (Tregs) by expression of *Foxp3* and *Il10*. In addition, we identified a memory-like CD4^+^ T cell population with high expression of memory T cell signature genes *Tcf7*, *Id3*, and *Izumo1r* (Fig. 3, B and C). Frequency analysis indicated that memory-like CD4^+^ IEL-T cells are the dominant CD4^+^ IEL-T cell subpopulation, and that CD4^+^ LPL-T cells have higher Th1 cell and Treg cell proportions and lower memory-like T cell proportion compared with CD4^+^ IEL-T cells (Fig. 3 D). It also suggested a dramatic increase in memory-like CD4^+^ T cells and Treg cells within epithelium layer and lamina propria of HFHS diet intestine. In contrast, the frequencies of Th1 cells decreased (Fig. 3 D). To further validate the existence and proportional change of identified Th1 cell and Treg cell populations, we utilized both intraepithelial and lamina propria immune cells from an additional cohort of mice (16 chow diet, 16 HFHS diet), and profiled intracellular IFNγ and Foxp3 expression. Flow cytometry analysis further supported the results from scRNA-seq, demonstrating that CD4^+^ IEL-T cell and CD4^+^ LPL-T cell decreased their Th1 cell proportions and increased Treg cell proportions in HFHS diet intestine (Fig. 3, E and F; and Fig. S3 E). Due to the huge level of total CD4^+^ T cell infiltration in the lamina propria upon HFHS diet feeding, we profiled the small intestines from chow diet fed mice and HFHS diet fed mice using RT-QPCR. Expression levels of *Ifng* indicate that there is a slight increase in Th1 responses at the level of the whole intestinal tissue (Fig. S3 F). Expression of *Il17a* and *Il17f* both dramatically increased in the HFHS diet fed mice (Fig. S3 F). Moreover, we used flow cytometry to further verify the changes in Th1 and Th17 responses; the absolute cell counting indicates that there is a slight increase in LPL-Th1 cells and a significant increase in LPL-Th17 cells in the intestine upon HFHS diet feeding (Fig. S3 G).

Although the cellular plasticity of CD4^+^ T cells in the intestine has been noted (Brucklacher-Waldert et al., 2014), the characterization and ontogeny of those subpopulations are not well understood. UMAP visualization of CD4^+^ T cell subsets suggested that the memory-like CD4^+^ T cell population is most transcriptionally related to both Treg and the Th1/Th2/Th17 clusters, whereas Th1, Th2, and Th17 clusters are close to each other (Fig. 3 A). CytoTRACE analysis indicated that memory-like T cell is the least differentiated cell status of CD4^+^ T cell in the intestine with the highest score, whereas Th1, Th2, Th17, and Treg cells represented highly differentiated cell status with mid to low scores, by which they form four independent columns paralleled with each other in the 3D plot, all related to the memory-like T cells at the top (Fig. 3 G). Projection of the velocity field arrows showed a strong directional flow of the memory-like T cells to both Treg fates and Th1/Th2/Th17 fates (Fig. 3 H). Monocle pseudotime analysis further corroborated the transitions from memory-like CD4^+^ cells to the Treg cells and Th1/Th2/Th17 cells (Fig. 3 I). Those results suggested that mouse intestine Treg and T helper cell populations might be continuously replenished from the memory-like T cell populations.

We then assessed the potential upstream regulators of the DEGs between memory-like CD4^+^ T cells and Th1, Th2, Th17, as well as Treg cells using IPA. The results suggested that some signaling pathways such as BHLHE40 and CTNNB1 (Wnt/β-catenin) are significant mediators in all the transitions from CD4^+^ memory-like T cells to Th1, Th2, Th17 cells and to Treg cells, and that some pathways such as RelA/p65 and TGFβ/KLF6 are involved in Th17 and Treg cell transitions (Fig. 3, J–M). Signature regulators were also observed in the transitions from CD4^+^ memory-like T cells to Th1 (TBX21, STAT4, STAT1), Th2 (STAT5A, STAT5B), Th17 (ID2, EP300, GLI3), and Treg (SMAD3, SMAD4) cells (Fig. 3, J–M; and Fig. S3 H). Thus, these results suggested that the HFHS diet microenvironment-induced shifts in intestine CD4^+^ T cell populations occurred both within epithelium layer and lamina propria, and that this might be influenced by the transition from CD4^+^ memory-like T cells toward Th1, Th2, Th17, and Treg cells.

### Distinct myeloid cell subsets accumulate in HFHS diet intestine

**Figure S4.**
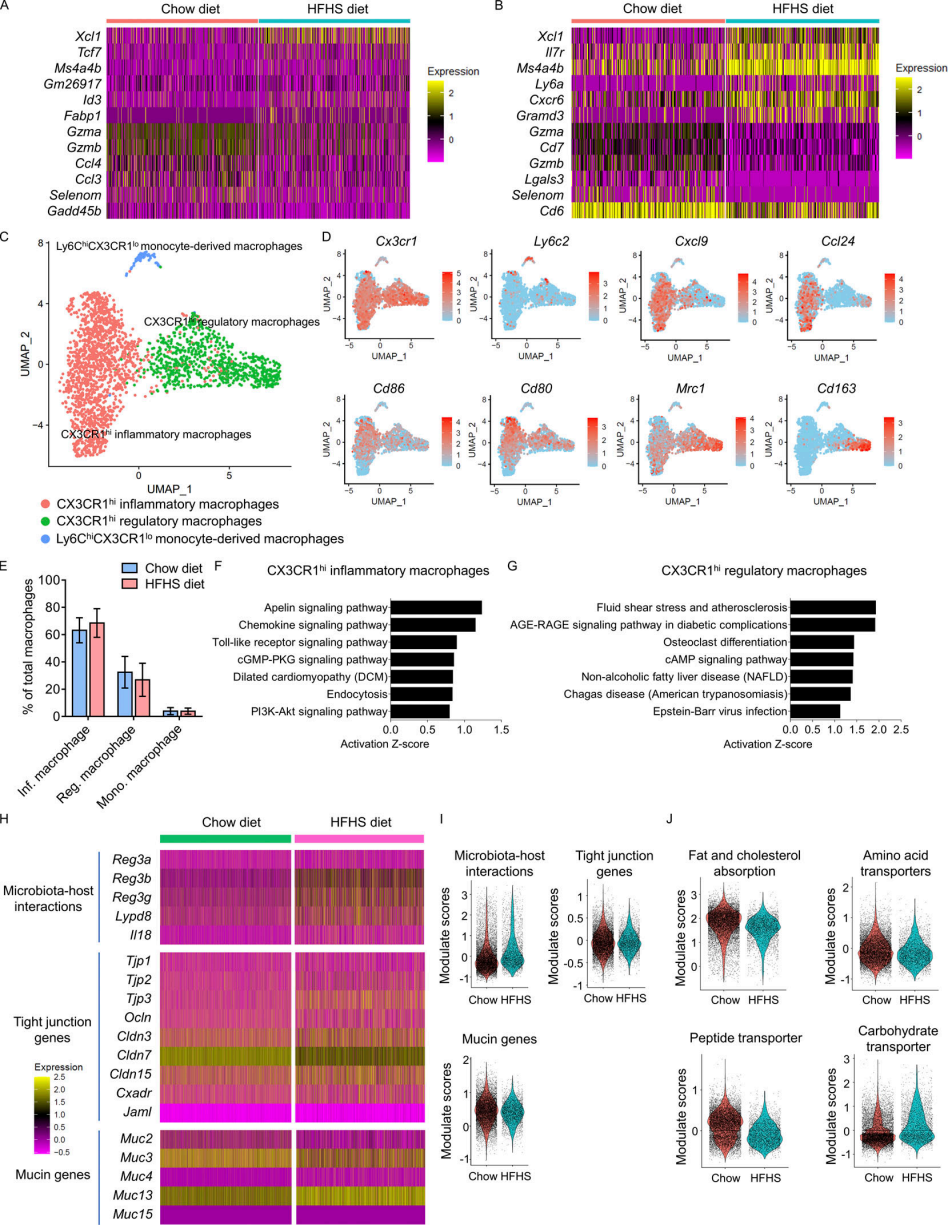
**Analysis of the macrophage populations and the comparison of key genes related to intestine barrier function and nutrient absorption function between chow diet and HFHS diet intestine. (A)** Heatmap showing the top six most upregulated and most downregulated DEGs in CD8αα^+^ IEL-T cells between chow diet intestine and HFHS diet intestine. **(B)** Heatmap showing the top six most upregulated and most downregulated DEGs in CD8αβ^+^ IEL-T cells between chow diet intestine and HFHS diet intestine. **(C–G)** An independent cohort consisting of 10 chow diet mice and 10 HFHS diet mice were added to the previous cohort (Fig. 1 A) to accumulate more macrophages. **(C)** UMAP visualization of mouse intestine macrophages derived from lamina propria of 20 chow diet mice and 20 HFHS diet mice is shown, where individual points correspond to single cells. Cluster analysis yields three distinct clusters: Ly6C^hi^CX3CR1^lo^ monocyte-derived macrophage (blue), CX3CR1^hi^ inflammatory macrophage (red), and CX3CR1^hi^ regulatory macrophage (blue). **(D)** Feature plots showing the selected signature genes projection on UMAP. Intensity of gene expression in each cell was indicated in color saturation. **(E)** Histogram showing the proportions of macrophages derived from 20 chow diet mice and 20 HFHS diet mice. Inf. macrophage, CX3CR1^hi^ inflammatory macrophage; Reg. macrophage, CX3CR1^hi^ regulatory macrophage; Mono. Macrophage, Ly6C^hi^CX3CR1^lo^ monocyte-derived macrophage. **(F and G)** KEGG pathway gene set enrichment analysis of DEGs between chow diet and HFHS diet mice intestine cells. The top seven most upregulated signaling pathways in response to the HFHS diet were shown. **(F)** CX3CR1^hi^ inflammatory macrophages. **(G)** CX3CR1^hi^ regulatory macrophages. **(H)** Heatmaps of selected genes that are involved in microbiota–host interactions, tight junction, and mucin functions. **(I)** Violin plots showing the average signature scores calculated for microbiota–host interactions, tight junction, and mucin functions. Each dot represents one cell. **(J)** Violin plots showing the average signature scores calculated for the absorption of distinct nutrient classes (fat and cholesterol, amino acid, peptide, and carbohydrate). Each dot represents one cell.

**Figure 4.**
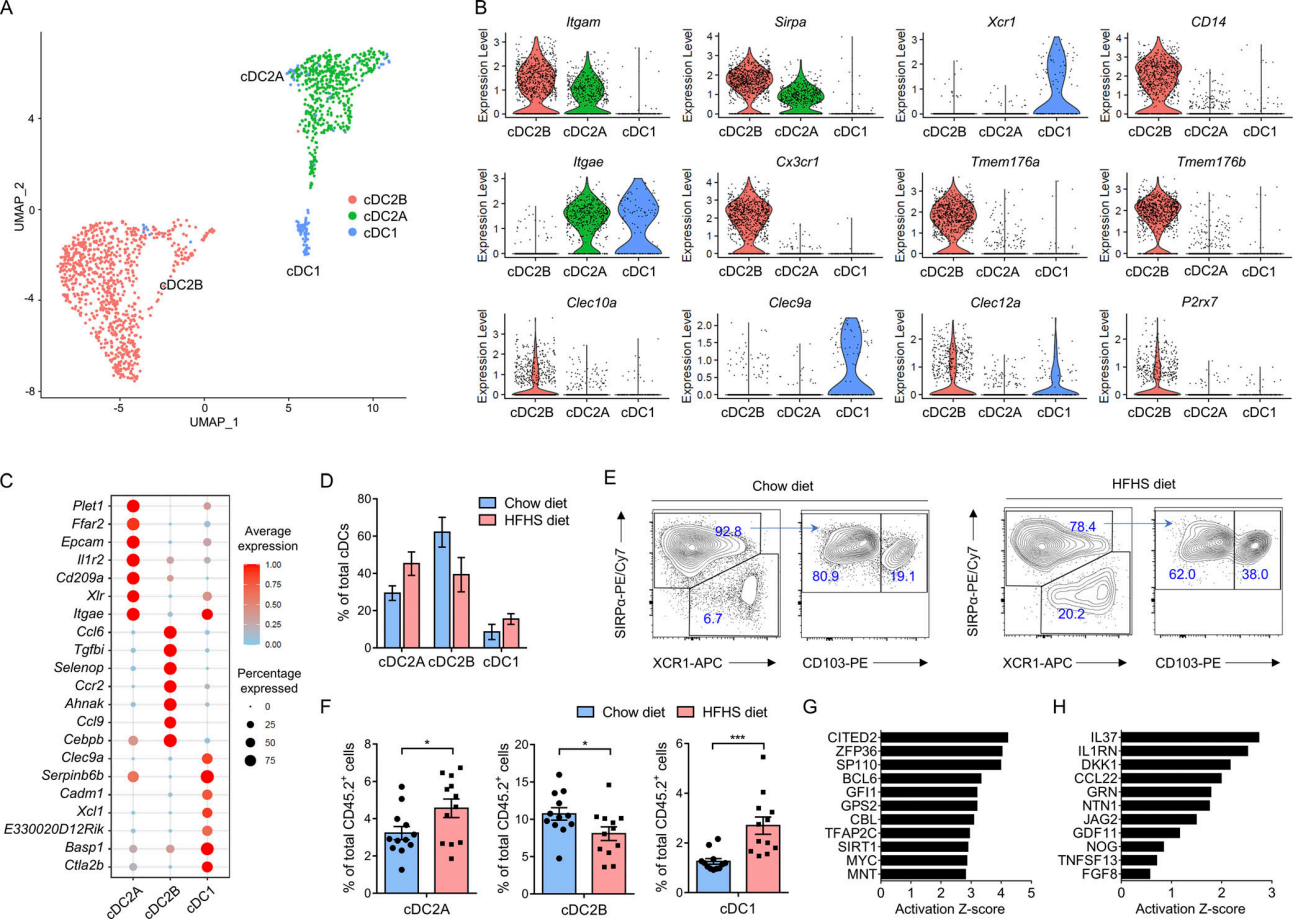
**Distinct dendritic cell subsets accumulate in HFHS diet intestine. (A and B)** An independent cohort consisting of 10 chow diet mice and 10 HFHS diet mice were added to the previous cohort (Fig. 1 A) to accumulate more cDCs. **(A)** UMAP visualization of mouse intestine cDCs derived from lamina propria of 20 chow diet mice and 20 HFHS diet mice is shown, where individual points correspond to single cells. Cluster analysis yields three distinct clusters: cDC2B (red), cDC2A (green), and cDC1 (blue). **(B)** Violin plots showing expression of the selected signature genes in cDC populations. Each dot represents one cell. **(C)** Dot plot showing selected top DEGs for the populations depicted in cDCs. Color saturation indicates the strength of average gene expression, whereas the dot size reflects the percentage of each cell cluster expressing the gene. **(D)** Histogram showing the proportions of cDCs derived from 20 chow diet mice and 20 HFHS diet mice. **(E and F)** Flow cytometry analyses of scRNA-seq-defined mouse cDC populations (gated as CD45.2^+^CD11b^low^CD11c^high^I-A/I-E^high^PDCA^−^ cells) in an additional cohort of 12 chow diet mice and 12 HFHS diet mice (E; Fig. S1 A). Histogram showing the proportions of cDC subpopulations (F). cDC1s, XCR1^+^SIRPα^−^; cDC2As, SIRPα^+^CD103^+^; cDC2Bs, SIRPα^+^CD103^−^. **(G and H)** IPA analysis of transcriptional upstream regulators (G) and secreted upstream regulators (H) of the transition of cDCs from chow diet to HFHS diet based on DEGs. Representative of 1 (A–D, G, and H), and 5 (E and F) experiments. All data are presented as the mean ± SEM. *P < 0.05, **P < 0.01, ***P < 0.001, and ****P < 0.0001 by Student’s *t* test (F). Statistics are all two-sided.

Classic/conventional dendritic cells (cDCs) are required for peripheral Treg induction in the intestine, mediate tolerance to food antigens, limit reactivity to the gut microbiota and optimize responses to intestinal pathogens (Cabeza-Cabrerizo et al., 2021; Esterházy et al., 2016; Worbs et al., 2017). Previous results indicated increased proportions of Treg cells in CD4^+^ IEL-T cells and CD4^+^ LPL-T cells, and increased proportions of memory-like T cells in both CD8αα^+^ IEL-T cells and CD8αβ^+^ IEL-T cells in response to the HFHS diet (Fig. 2, D and E; and Fig. 3, D and E). Notably, memory-like T cells are characterized by *Xcl1* signature gene expression (Fig. 2 C), and *Xcl1* is the most differentially expressed gene in both CD8αα^+^ IEL-T cells and CD8αβ^+^ IEL-T cells when two diets were compared (Fig. S4, A and B; and Tables S1 and S2). The receptor for XCL1, XCR1, is known to be exclusively expressed by cDC1 and conserved across species (Cabeza-Cabrerizo et al., 2021; Worbs et al., 2017). To examine whether further cellular heterogeneity existed in cDCs, an additional cohort of 26,848 lamina propria immune cells from 10 HFHS diet mice and 10 chow diet mice were profiled by scRNA-seq. Assessment of pooled cDCs from all samples revealed three distinct populations (Fig. 4 A). cDC1 is discerned by expression of *Xcr1*, *Itgae* (CD103), and *Clec9a*. cDC2B is distinguished by expression of *Itgam* (CD11b), *Sirpa* (SIRPα), *Cd14*, *Cx3cr1*, *Tmem176a*, *Tmem176b*, *Clec10a*, *Clec12a*, *P2rx7*, and lack of *Itgae*. cDC2A is identified via *Itgam*, *Sirpa*, *Itgae*, and lack of expression of cDC2a markers such as *Clec10a* and *P2rx7* (Fig. 4, B and C). Frequency assessments indicated a decrease in cDC2B proportion in HFHS diet intestine, whereas the frequencies of cDC2A and cDC1 both increased (Fig. 4 D). An additional cohort of mice (12 chow diet, 12 HFHS diet) was profiled by flow cytometry to further confirm the existence and proportional change of identified cDCs (Fig. S2 D). This corroborated the results from the scRNA-seq and identified three distinct cDC populations based on the expression of SIRPα, XCR1, and CD103 (Fig. 4 E). Flow cytometry also demonstrated the increase of cDC2A and cDC1 proportions and decrease of cDC2B proportions in HFHS diet intestine, consistent with scRNA-seq analysis (Fig. 4 F).

Next, we sought to investigate the potential molecular mechanisms regulating the intestine cDC alteration. We assessed the upstream regulators of the DEGs between all chow diet cDCs and HFHS diet cDCs utilizing IPA. The results indicated that upstream transcription factors CITED2 and ZFP36 are at the top of the list (Fig. 4 G). They are involved in TGFβ signaling pathway activation and NF-κB inhibition, and are known for restraining pathogenic proinflammatory gene programs of myeloid cells and promoting the immunoregulatory functions (Makita et al., 2021; Pong Ng et al., 2020). Further analysis of the secreted upstream regulators showed that cytokines and growth factors, such as IL-37, IL1RN, and DKK1, play a critical role in cDC transformation in response to HFHS diet feeding (Fig. 4 H), presumably by negatively regulating the proinflammatory activation of cDCs and facilitating Treg induction (Chu et al., 2021; Dinarello et al., 2016; Li et al., 2015). These data indicate a potential immunoregulatory transformation of cDCs in the intestine upon HFHS diet feeding and identify enhanced signaling pathways such as IL-37, TGFβ (CITED2), and inhibited pathways such as NF-κB (ZFP36), IL-1β (IL1RN), and Wnt (DKK). Collectively, these signaling pathways are likely to play critical roles in the cDC transformation and subset regulation.

Furthermore, we also examined whether further cellular heterogeneity existed in macrophages. We assessed pooled macrophages from all lamina propria samples and UMAP visualization revealed three distinct populations (Fig. S4 C). Besides the identification of Ly6C high (Ly6C^hi^) CX3CR1 low (CX3CR1^lo^) monocyte-derived macrophages, we found two major heterogeneous populations in the macrophages with high CX3CR1 expression: CX3CR1^hi^ inflammatory macrophages are discerned by expression of *Cd86*, *Cd80*, *Ccl24*, and *Cxcl9*; CX3CR1^hi^ regulatory macrophages are distinguished by expression of *Mrc1* (CD206) and *Cd163* (Fig. S4 D). Frequency analysis suggested a slight increase in the percentage of CX3CR1^hi^ inflammatory macrophages and a decrease in the proportion of CX3CR1^hi^ regulatory macrophages in the HFHS diet intestine, while Ly6C^hi^CX3CR1^lo^ monocyte-derived macrophages remain unchanged (Fig. S4 E). We compared the differentially expressed genes for the populations depicted in the macrophages. The results indicate that CX3CR1^hi^ inflammatory macrophages express high levels of co-stimulatory ligands CD86 and CD80, and chemokine CXCL9, which are critical for T cells activation and recruitment (Table S3). CX3CR1^hi^ regulatory macrophages express high levels of immunoregulatory genes, such as *Mrc1* and *Clec12a*, and also produce growth factors such as IGF-binding proteins (*Igfbp4*) and growth and differentiation factor 15 (*Gdf15*), which could further regulate the whole-body metabolism through the circulation (Table S3). Ly6C^hi^CX3CR1^lo^ monocyte-derived macrophages express high levels of *Ly6c2* and activation marker *Cd69*, indicating their early activation and maturation status (Table S3). Furthermore, we also performed the KEGG pathway enrichment analysis of two major macrophage subpopulations. Analysis of CX3CR1^hi^ inflammatory macrophages in HFHS diet intestine showed a strong upregulation in chemokine, Toll-like receptor and PI3K-Akt signaling pathways, indicating their enhanced proinflammatory status (Fig. S4 F). Analysis of CX3CR1^hi^ regulatory macrophages also showed increased KEGG pathways involved in diseases such as atherosclerosis, diabetic complications, and NAFLD (Fig. S4 G). These results suggest that both CX3CR1^hi^ inflammatory macrophages and CX3CR1^hi^ regulatory macrophages play important roles in the activation and regulation of immune responses in the intestine upon HFHS diet feeding, and CX3CR1^hi^ regulatory macrophages might also be involved in the modulation of systemic metabolic disorders when fed with a HFHS diet.

### Diversity in structural cells and altered nutrient absorption uncovered

**Figure 5.**
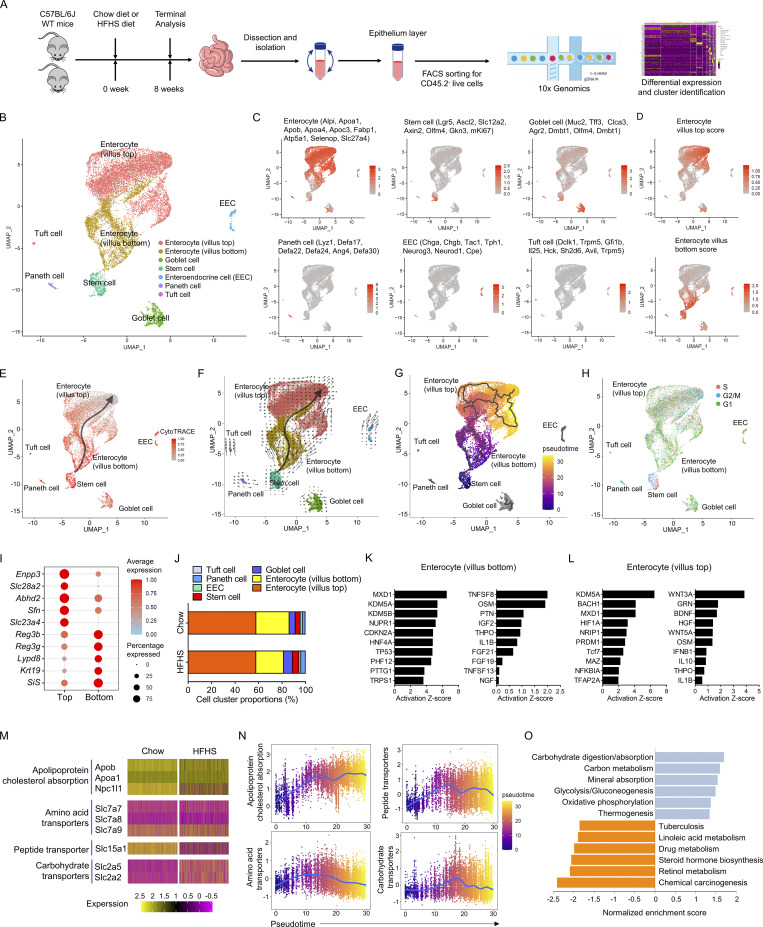
**Diversity in structural cells and altered nutrient absorption uncovered. (A)** Schematic of the experimental pipeline. C57BL/6J mice were fed on chow diet and HFHS diet for 8 wk before dissection and small intestines were isolated. Cells from epithelium layer were sorted for CD45.2^−^ live cells and analyzed using 10× Genomics Chromium droplet scRNA-seq. Cells were clustered via differential gene expression for further studies. EEC, enteroendocrine cell. **(B)** UMAP plot of IECs derived from a cohort of 10 chow diet mice and 10 HFHS diet mice, where individual points correspond to single cells. Cluster analysis yields seven distinct clusters comprising IECs. **(C and D)** Feature plots showing the mean expression of known marker genes for a particular cell type or state projected on UMAP. Average scores in each cell are indicated in color saturation. **(C)** Average scores of signature genes for cluster annotation of enterocyte, stem cell, goblet cell, Paneth cell, EEC, and tuft cell (genes indicated above each plot). **(D)** Average scores of signature genes cluster annotation of enterocyte on top of the villus or at the bottom of the villus (genes indicated in Tables S4 and S5). **(E)** Visualizing the predicted differentiation using CytoTRACE analysis of IECs. The color indicates the levels of CytoTRACE score from 1 (red, lowest levels of differentiation) to 0 (grey, highest levels of differentiation). Feature plots showing the CytoTRACE score projections on UMAP plot. **(F)** RNA-velocity analysis of IECs with velocity field projected onto the UMAP plot. Arrows show the local average velocity evaluated on a regular grid and indicate the extrapolated future states of cells. **(G)** Monocle analysis of the IECs. The color indicates pseudotime directionality projection on UMAP from the earliest (blue) to the latest (yellow). **(H)** Cell cycle analysis of the IECs. Predicted classification of each cell in either G2/M (red), S (blue), or G1 (green) phase was projected on UMAP. **(I)** Dot plot showing the top DEGs for the enterocyte (villus top) and enterocyte (villus bottom) populations depicted. Color saturation indicates the strength of average gene expression, whereas the dot size reflects the percentage of each cell cluster expressing the gene. Top, enterocyte (villus top); bottom, enterocyte (villus bottom). **(J)** Histogram showing the proportions of IECs derived from 10 chow diet mice and 10 HFHS diet mice. **(K and L)** IPA analysis of transcriptional upstream regulators (left) and secreted upstream regulators (right) of the transition from stem cell to enterocyte (villus bottom; K) or enterocyte (villus bottom) to enterocyte (villus top; L) based on DEGs. **(M)** Heatmaps of selected genes that are critical for the absorption of distinct nutrient classes. **(N)** Average scores of critical genes expression for the absorption of distinct nutrient classes (fat and cholesterol, amino acid, peptide, and carbohydrate) in each cell from stem cells in the crypt to the enterocytes on the top of the villus. Zonation of the cells along the villus length was profiled by the pseudotime trajectories from Monocle analysis from the earliest (blue) to the latest (yellow; Fig. 5 F). **(O)** KEGG pathway gene set enrichment analysis of DEGs between chow diet IECs and HFHS diet IECs, indicating the top six-most upregulated (top) and most downregulated signaling pathways (bottom) in response to the HFHS diet.

The intestinal epithelium is composed of a single layer of IECs. Although IECs are non-hematopoietic structural cells, they are an integral component of intestinal immunity and play important roles in the absorption of nutrients, protection of mucosal barrier functions, and maintenance of intestinal homeostasis (Allaire et al., 2018; Peterson and Artis, 2014). However, how IECs are modulated by the intestine microenvironment in response to a HFHS diet is poorly understood. We sorted non-hematopoietic CD45.2^−^ live cells from epithelium layer of a new cohort of 10 chow diet mice and 10 HFHS diet mice, and then profiled them using scRNA-seq (Fig. 5 A). The resulting quality-controlled, mouse intestinal structural cell atlas included 13,664 cells, which were clustered based on differential expression of hallmark genes and visualized using UMAP: enterocytes by expression of *Alpi*, *Apoa1*, *Apob*, *Apoa4*, *Apoc3*, *Fabp1*, *Atp5a1*, *Selenop*, and *Slc27a4*; stem cells by expression of *Lgr5*, *Ascl2*, *Slc12a2*, *Axin2*, *Olfm4*, *Gkn3*, and *mKi67*; goblet cells by expression of *Muc2*, *Tff3*, *Clca3*, *Agr2*, *Dmbt1*, *Olfm4*, and *Dmbt1*; Paneth cells by expression of *Lyz1*, *Defa17*, *Defa22*, *Defa24*, *Ang4*, and *Defa30*; enteroendocrine cells (EECs) by expression of *Chga*, *Chgb*, *Tac1*, *Tph1*, *Neurog3*, *Neurod1*, and *Cpe*; and tuft cells by expression of *Dclk1*, *Trpm5*, *Gfi1b*, *Il25*, *Hck*, *Sh2d6*, *Avil*, and *Trpm5* (Fig. 5, B and C). The enterocytes include heterogeneous subclusters that could be further distinguished by zonation through modulation scores of the top landmark and bottom landmark genes based on their distribution along the intestinal villus axis (Fig. 5, B and D; and Tables S4 and S5; Moor et al., 2018). UMAP visualization of IEC clusters showed that the intestine epithelial stem cells are transcriptionally close to enterocytes (villus bottom) populations, and the enterocytes (villus bottom) appeared to be most transcriptionally related to stem cell and enterocyte (villus top) populations at the same time (Fig. 5 B). CytoTRACE analysis indicated that stem cells are the least differentiated cells and enterocytes (villus top) are highly differentiated, whereas enterocyte (villus bottom) represents a less differentiated cell status in the middle (Fig. 5 E). Projection of the velocity field showed a strong directional flow of stem cell to enterocyte (villus bottom) and then enterocyte (villus top) fates (Fig. 5 F). Monocle analysis indicated a strong developmental trajectory from stem cell towards enterocyte (villus top), with enterocyte (villus bottom) representing an additional transition state (Fig. 5 G). Cell cycle analysis further validated stem cell status through a high proportion of cells in either G2/M or S phase (Fig. 5 H). These data indicated the heterogeneity of enterocytes along the developmental path from the bottom to the top of the villi.

The top variable expressed genes indicated that enterocytes (villus top) have higher expression of genes that facilitate nutrient transportation, while enterocytes (villus bottom) express higher levels of genes for antimicrobial and immunoregulatory components (Fig. 5 I). We further examined the key functional genes related to intestinal homeostasis and barrier function. Although the enterocyte (villus bottom) proportion decreased slightly (Fig. 5 J), the expression of genes involved in microbiota-host interactions dramatically increased in HFHS diet intestine. The expression of tight junction genes and mucin genes remains unchanged (Fig. S4, H and I). IPA analysis revealed dramatic differences in upstream signaling regulators between the transition from stem cell to the enterocyte (villus bottom) status and the subsequent transition to the enterocyte (villus top) status (Fig. 5, K and L). Notably, transcription factors such as MXD1 and KDM5A promote enterocyte maturation across the development trajectory. Growth factors such as oncostatin M (OSM), pleiotrophin (PTN), and TNFSF8 promote the early development of enterocytes at the bottom of the villus, whereas the WNT (WNT3A, WNT5A) and granulin (GRN) signaling pathways are crucial for the maturation of enterocytes during their migration from the bottom of the villus to the top (Fig. 5, K and L).

A fundamental role of IECs is the absorption of nutrients, by which the IEC and whole-body metabolism are tightly interrelated (Zietek et al., 2020). Hence, we examined the transporters for key nutrient families and found that carbohydrate transporters were enriched in the HFHS diet IECs, whereas the expression of the *Slc15a1* gene that encodes the main peptide transporter Pept1, the cholesterol transporter Npc1l1 and the expression of genes for the lipoprotein biosynthesis machinery necessary for the assembly of chylomicrons were all decreased (Fig. 5 M and Fig. S4 J). We also noted that the transporters for these key nutrient families have their own expression domains along the pseudo-timeline from stem cells to the enterocyte (villus top). The amino acid and carbohydrate transporters were enriched at the bottom to the middle of the villi, whereas peptide transporters shifted in expression towards the upper villus zones, and the lipid transporters peaked in expression at the villi tips (Fig. 5, G and N). Furthermore, the KEGG pathway enrichment analysis indicated a strong upregulation in carbohydrate absorption and carbon metabolisms in HFHS diet IECs (Fig. 5 O). In sum, single-cell profiling of IECs revealed heterogeneity in enterocytes, distinct transporter zonation and unique metabolic adaptions in response to the HFHS diet.

### Ligand–receptor analysis reveals intestine interactome network

**Figure 6.**
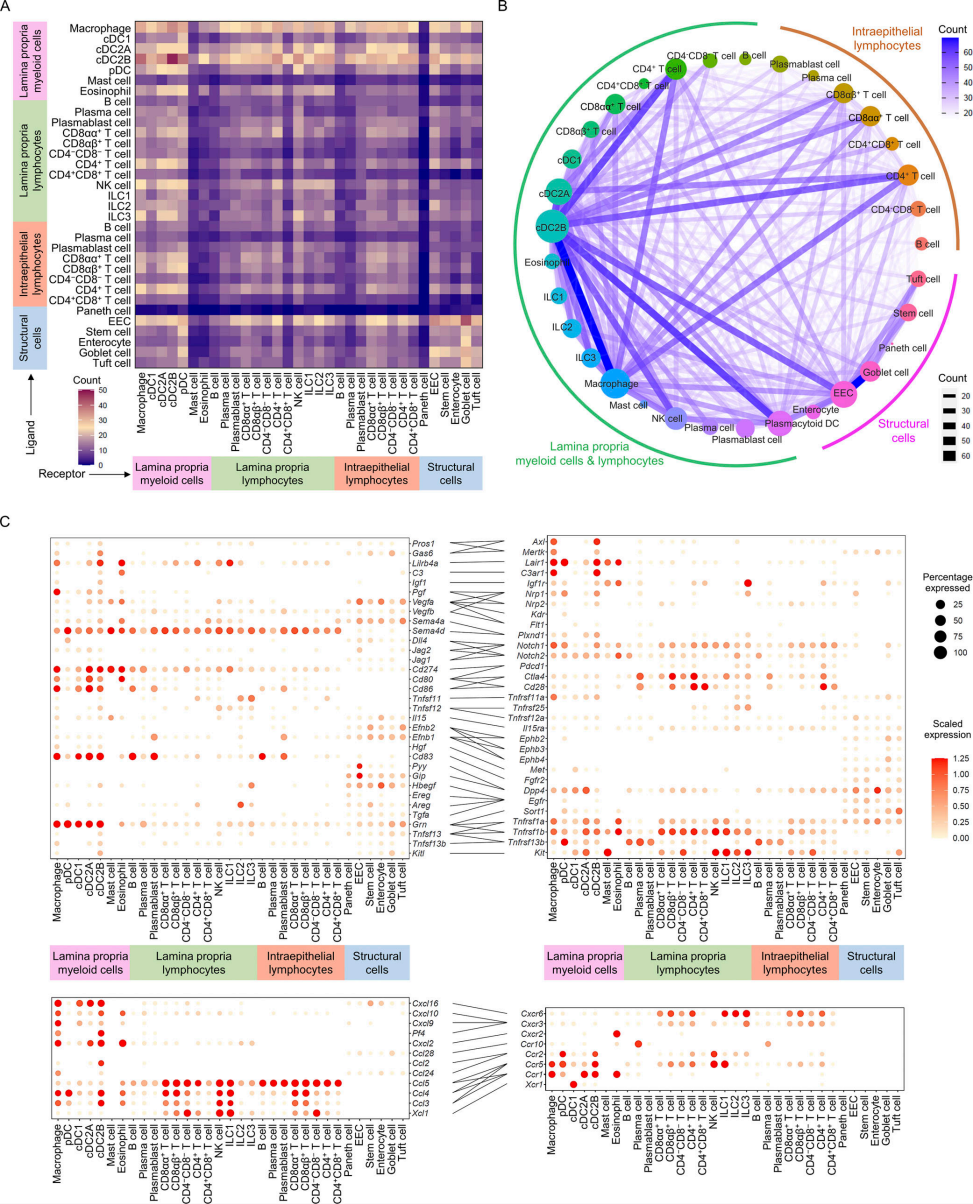
**Ligand–receptor analysis reveals intestine interactome network. (A)** Interaction heatmap plotting the total number of chow diet intestinal cell ligand (y axis) and receptor (x axis) interactions for the specified cell types based on expression of the gene in at least 10% of the cell population. The color represents the number of interactions between cell types: higher number of interactions (red), lower number of interactions (blue). **(B)** Connectome web analysis of chow diet intestine highly interacting cell types based on ligand–receptor interactions. Vertex (colored node of specific cell type) size is proportional to the number of interactions to and from that cell type, whereas the thickness of the connecting lines is proportional to the number of interactions between two nodes. **(C)** Dot plots showing expression of ligands (left) and receptors (right) in chow diet mouse intestinal cells. Implicated chemokines are shown in the lower panel. Color saturation in dot indicates the strength of expression in specific cell type, whereas dot size reflects the percentage of cells in each population expressing the gene.

In order to investigate how intestine cellular communication networks and context-dependent crosstalk of different cell types enable diverse physiological processes to proceed in the intestine, we performed ligand–receptor analysis on all major intestinal cell populations from chow diet intestine using CellPhoneDB to generate intestine homeostatic interactomes (Efremova et al., 2020). This revealed tens of thousands of potential structural cell-to-immune cell, immune cell-to-immune cell, as well as structural cell-to-structural cell interactions (Tables S6 and S7). All of the interactions were further verified using CellTalkDB, a manually curated database of literature-supported ligand–receptor interactions (Shao et al., 2021). The interactomes indicated that lamina propria myeloid cells are major signal sources. There are strong interactions between myeloid cells and lymphocytes, as well as myeloid cells and structural cells. The strongest interactions were observed within myeloid cells and within structural cells, while moderate levels of interactions were observed within lymphocytes (Fig. 6 A). Connectome web analysis of the chow diet intestine revealed that cDC2B and macrophage are central communication hubs among immune cells. Not only do they have strong interactions between themselves, but also with pDCs, CD4^+^ LPL-T cells, CD8αα^+^ IEL-T cells, CD8αβ^+^ IEL-T cells, and EECs (Fig. 6 B). Notably, EEC is a central communication hub among structural cells and strong interactions were observed with the goblet cells (Fig. 6 B).

The analysis implicated a variety of uncharacterized as well as validated signaling pathways in intestinal homeostasis. The chow diet intestine interactome suggested that cDC2Bs serve an immunoregulatory role in the chow diet intestine via the production of protein S (*Pros1*), growth arrest-specific 6 (*Gas6*), complement component 3 (*C3*), leukocyte immunoglobulin-like receptor, subfamily B, member 4 (LILRB4; *Lilrb4a*), CD83 (*Cd83*), insulin-like growth factor 1 (*Igf1*), and placenta growth factor (*Pgf*). The interactions were not only with immune cells like macrophages, pDCs, mast cells and eosinophils, but also cDC2B itself. These immune cells further contribute to the regulation of the IEL-T cells and LPL-T cells via interactions involving Jagged2 (*Jag2*), and delta-like 4 (*Dll4*), B7-1 (*Cd86*), B7-2 (*Cd80*) and PD-L1 (*Cd274*). Meanwhile, semaphorin 4D (*Sema4d*), CCL3, CCL4, and CCL5 signals sent out by intraepithelial and lamina propria lymphocytes could affect macrophages, pDCs, cDC2As, and cDC2Bs in return (Fig. 6 C). As a major signal source of structural cells, EECs play an immunoregulatory role and interact with macrophages, pDCs, cDC2As, and cDC2Bs via vascular endothelial growth factor A (*Vegfa*) and semaphorin 4A (*Sema4a*) signals, which are crucial for maintaining T-cell priming function in dendritic cells and promoting the stability and function of Treg cells (Delgoffe et al., 2013). EEC is also the major source of gastric inhibitory polypeptide (*Gip*) and pancreatic polypeptide (*Ppy*), which play critical roles in intestinal homeostasis and epithelium integrity (Adriaenssens et al., 2015). As a major signal receiver among structural cells, Goblet cells receive signals via ephrin B1 (*Efnb1*), ephrin B2 (*Efnb2*), IGF1, hepatocyte growth factor (*Hgf*), and epidermal growth factor receptor ligands (*Hbegf*, *Ereg*, *Areg*, and *Tgfa*) from other structural cells and granulin (*Grn*) from macrophages, pDCs, and cDCs (Fig. 6 C). ILC2 and ILC3 are the major sources of granulocyte-macrophage colony-stimulating factor (GM-CSF; *Csf2*), indicating their indispensable role in dendritic cell development and function. Macrophage and cDC2B are the major sources of CXCL16, CXCL10, CXCL9, and platelet factor 4 (*Pf4*), indicating their roles in the recruitment and maintenance of IELs and LPLs in the intestine. EECs and goblet cells interact with all major immune cell populations including myeloid cells, plasma cells, ILCs, IEL-T cell, and LPL-T cell via expression of CX3CL1, CCL28, and CCL25, suggesting their additional roles in immune cell recruitment and maintenance of overall immune homeostasis (Fig. 6 C).

### Dramatic remodeling of the intestine interactome network in response to the HFHS diet

**Figure 7.**
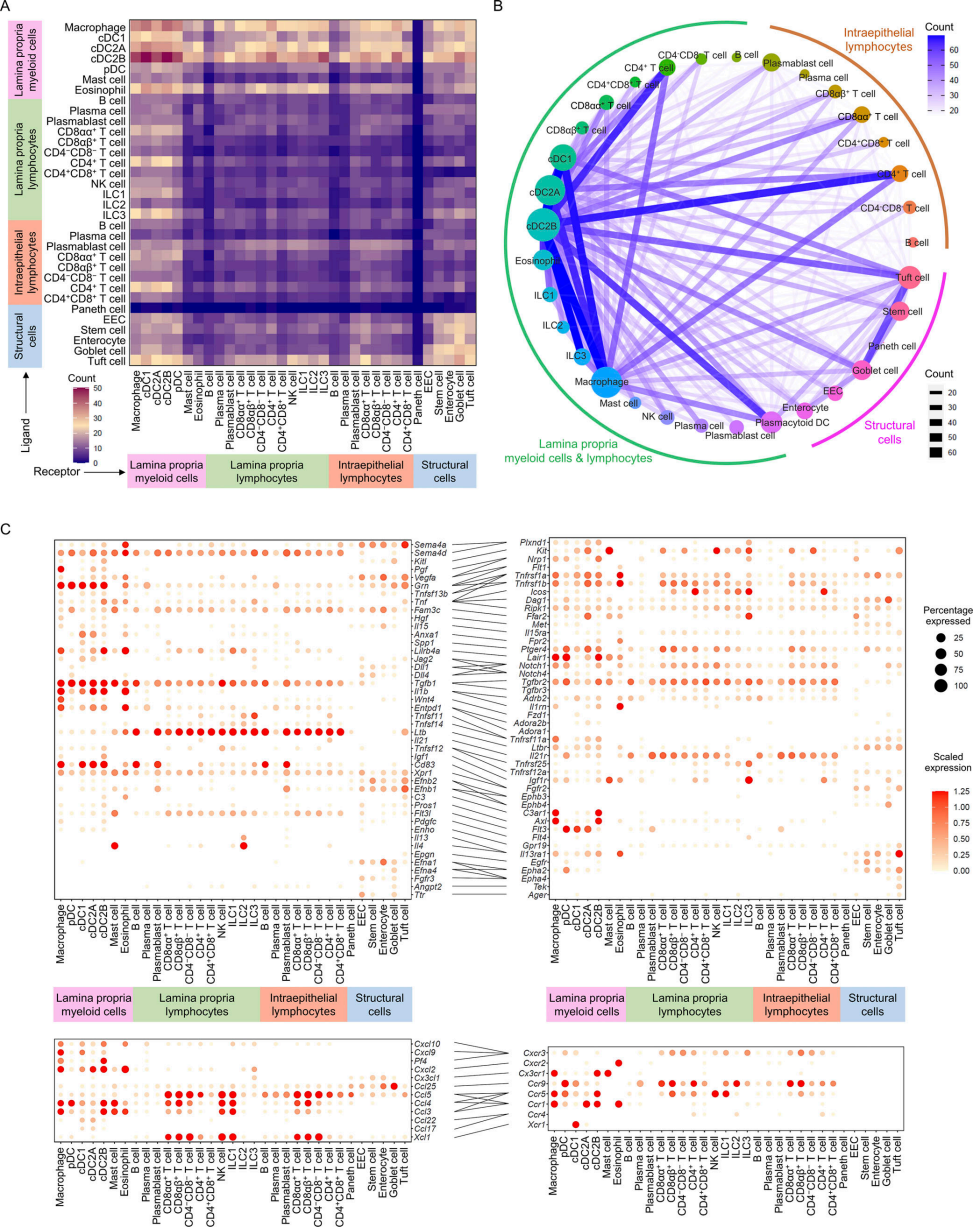
**Dramatic remodeling of the intestine interactome network in response to the HFHS diet. (A)** Interaction heatmap plotting the total number of HFHS diet intestinal cell ligand (y axis) and receptor (x axis) interactions for the specified cell types based on expression of the gene in at least 10% of the cell population. The color represents the number of interactions between cell types: higher number of interactions (red), lower number of interactions (blue). **(B)** Connectome web analysis of HFHS diet intestine highly interacting cell types based on ligand–receptor interactions. Vertex (colored node of specific cell type) size is proportional to the number of interactions to and from that cell type, whereas the thickness of the connecting lines is proportional to the number of interactions between two nodes. **(C)** Dot plots showing expression of ligands (left) and receptors (right) in HFHS diet mouse intestinal cells. Implicated chemokines are shown in the lower panel. Color saturation in dot indicates the strength of expression in specific cell type, whereas dot size reflects the percentage of cells in each population expressing the gene.

**Figure S5.**
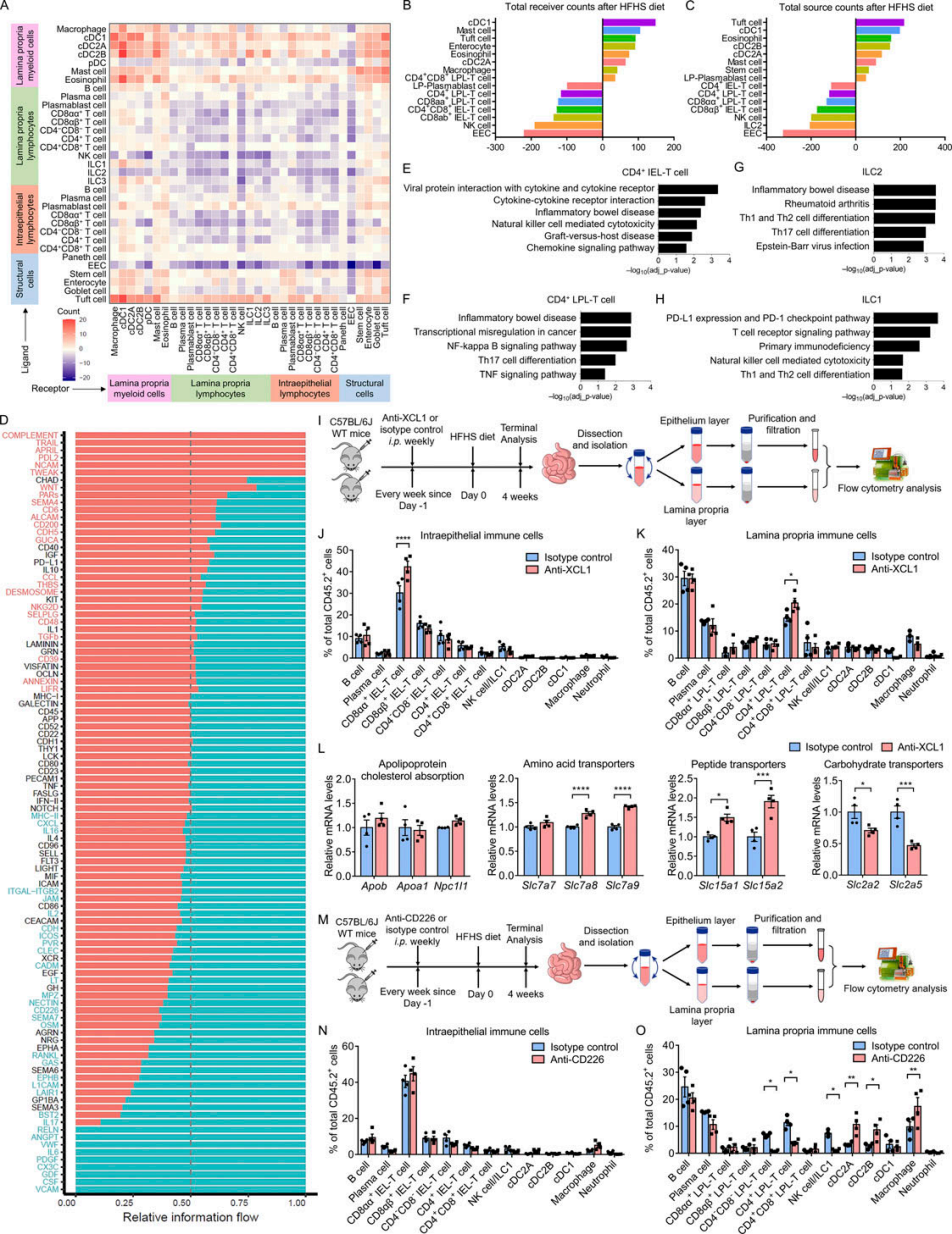
**Comparison between chow diet and HFHS diet intestine interactomes and antibody blockade experiments in vivo. (A)** Interaction heatmap showing the alternation of total number of interactions from chow diet intestine to HFHS diet intestine for the specified cell types based on expression of the gene in at least 10% of the cell population. Source cells, y axis; receiver cells, x axis. The color represents the number of interactions changes: higher number of interactions in HFHS intestinal cells (red), lower number of interactions in HFHS intestinal cells (blue). **(B)** Histogram showing the cell types most upregulated and most downregulated in total receiver counts from chow diet intestine to HFHS diet intestine. Each color represents a cell type. **(C)** Histogram showing the cell types most upregulated and most downregulated in total source counts from chow diet intestine to HFHS diet intestine. Each color represents a cell type. **(D)** Significant signaling pathways were ranked based on differences in the overall information flow within the inferred networks between chow diet intestine and HFHS diet intestine. The overall relative information flow of a signaling network is calculated by summarizing all communication probabilities in that network. The top signaling pathways with P value <0.05 were labeled with red (enriched in HFHS diet intestine) and blue (enriched in the chow diet intestine). A paired Wilcoxon test is utilized to determine whether there is significant difference between two datasets. IGF, insulin-like growth factor; GDF, growth differentiation factor; PDGF, platelet-derived growth factor; EGF, epidermal growth factor; EPHA, ephrin-A; EPHB, ephrin-B; APRIL, a proliferation-inducing ligand; SEMA, semaphorin; MPZ, myelin protein zero; GH, growth hormone; CEACAM, carcinoembryonic antigen-related cell adhesion molecules; RELN, reelin; TWEAK, TNF-related weak inducer of apoptosis; NRG, neuregulins; SELL, selection; LIFR, LIF receptor; ANGPT, angiopoietin; OSM, oncostatin M; ALCAM, activated leukocyte cell adhesion molecule; THBS, thrombospondin; JAM, junctional adhesion molecules; LT, lymphotoxin; PARs, protease-activated receptors; SELPLG, selectin P ligand; CLEC, C-type lectin-like receptor; MIF, macrophage migration inhibitory factor. **(E–H)** Top differentially regulated signaling pathways suggested in g:GOSt functional enrichment analysis of HFHS diet-enriched DEGs for the indicated cell types. **(E)** g:GOSt analysis of CD4^+^ IEL-T cell. **(F)** g:GOSt analysis of CD4^+^ LPL-T cell. **(G)** g:GOSt analysis of ILC2. **(H)** g:GOSt analysis of ILC1. **(I–K)** C57BL/6J mice received i.p. injection of anti-XCL1 antibodies (25 μg/animal, once per week; *n* = 4) to block XCL1–XCR1 signal; mice received i.p. injection of isotype antibodies (25 μg/animal, once per week; *n* = 4) were included as controls. Mice were fed on a HFHS diet for 4 wk and, following euthanasia, small intestines were isolated. Cells from the epithelium layer and lamina propria layer, were purified and then subjected to flow cytometry analysis. WT, wild type. **(I)** Schematic of the experimental pipeline. **(J and K)** Histogram showing the proportion of intraepithelial immune cells (J) and lamina propria immune cells (K) derived from mice intestine. **(L)** C57BL/6J mice received i.p. injection of anti-XCL1 antibodies (25 μg/animal, once per week; *n* = 4) to block XCL1–XCR1 signal; mice received i.p. injection of isotype antibodies (25 μg/animal, once per week; *n* = 4) were included as controls. Mice were fed on a HFHS diet for 4 wk and, following euthanasia, small intestines were isolated. Cells from epithelium layer were sorted for CD45.2^−^ live cells and analyzed using RT-QPCR. Histogram showing the mRNA expression levels of genes involved in fat and cholesterol absorption, amino acid transporters, peptide transporters, and carbohydrate transporters in sorted IECs. **(M–O)** C57BL/6J mice received i.p. injection of anti-CD226 antibodies (25 μg/animal, once per week; *n* = 4) to block NECTIN-CD226 signal; mice received i.p. injection of isotype antibodies (25 μg/animal, once per week; *n* = 4) were included as controls. Mice were fed on a HFHS diet for 4 wk and, following euthanasia, small intestines were isolated. Cells from epithelium layer and lamina propria layer, were purified and then subjected to flow cytometry analysis. **(M)** Schematic of the experimental pipeline. **(N and O)** Histogram showing the proportion of intraepithelial immune cells (N) and lamina propria immune cells (O) derived from mice intestine. Representative of 2 (I–O) experiments. All data are presented as the mean ± SEM. *P < 0.05, **P < 0.01, ***P < 0.001, and ****P < 0.0001 by two-way ANOVA (J–L, N, and O). Statistics are all two-sided.

To explore the changes that occurred to the intestine communication networks in response to a HFHS diet, we performed CellPhoneDB ligand–receptor analysis on all HFHS-diet intestinal cells and verified the identified interactions using CellTalkDB. The interactome analyses indicated an increased number of ligand–receptor pairings in myeloid cells in HFHS diet intestine, especially interactions involving cDC1 cells, while there were significantly decreased interactions between lymphocytes. Notably, EECs dramatically decreased their cell–cell interactions in HFHS diet intestine while tuft cells became the major signal source among all structural cells (Fig. 7 A; and Fig. S5, A–C). Connectome web analysis further revealed an enrichment of cDC1, cDC2B, macrophage, and tuft cell communication hubs within HFHS diet intestine (Fig. 7 B).

Intestine interactomes analyses showed that cDC1s have increased interactions with macrophages, cDC2As and cDC2Bs, and are the major receivers of the immune-regulatory signal of Wnt4, HGF, and PGF (Albonici et al., 2019; Haseeb et al., 2019; Hübel and Hieronymus, 2015). Further analysis in HFHS diet intestine identified cDC1 as a key regulator expressing immune-regulatory signals such as TGFβ (*Tgfb1*), ephrin B1, IL-15, annexin A1 (*Anxa1*), secreted phosphoprotein 1 (*Spp1*), and LILRB4. Our interaction analyses also indicated that both CD8αα^+^ T cells and CDαβ^+^ T cells dramatically increased the production of the chemokine XCL1 and were involved in the XCL1–XCR1-mediated recruitment of cDC1s, and that cDC1s further interact with CD4^+^ IEL-T cells, CD4^+^ LPL T cells, and ILC2s via CCL22 (Fig. 7 C; and Fig. S4, A and B). Analysis of the intestine interactome suggested that among myeloid cells, cDC2Bs and eosinophils may contribute to tissue inflammation during HSHS diet via TNF-, IL-1β (*Il1b*)-, KIT-ligand (*Kitl*)-, TNFSF13B-, TNFSF3 (*Ltb*)-, granulin-, semaphorin 4A-, CXCL10-, and CXCL2-mediated regulation of IEL-T cells, LPL-T cells, lamina propria ILCs, and myeloid subsets. The interactome also identified CD4^+^ IEL-T cells, CD4^+^ LPL T cells, ILC1s, ILC2s, and ILC3s as major producers of strong pro-inflammatory signals, including Fam3c, TNF, TNFSF3, TNFSF11, TNFSF14, and IL-21 (Fig. 7 C). Together with the increased proportions of CD4^+^ IEL-T cells, CD4^+^ LPL T cells and ILCs in HFHS diet intestine (Fig. 1, D and E), these results further supported their roles in the potentiation of inflammatory responses during HFHS diet feeding.

The chemokine analysis suggests that CCL3, CCL4, CCL5, and their main receptors (CCR1 and CCR5) play an important role in cDC2A and cDC2B recruitment. cDC2Bs also express high levels of CX3CR1, which receive signals from CX3CL1 produced by IECs (Fig. 7 C). As to the ILCs, NK cells and ILC1s receive signals from CCR5 ligands (CCL3, CCL4, and CCL5) due to the high expression of *Ccr5* gene. ILC1s, ILC2s, and ILC3s have high expression of CCR9, which could receive a signal from CCL25 produced by the IECs (Fig. 7 C). cDC1s and CD4^+^ LPL-T cells also express high levels of CCR9, indicating that IECs, especially goblet cells and enterocytes, play an important role in immune cell infiltration through the CCL25–CCR9 axis. CD4^+^ LPL-T cells, cDC1, NK cell, ILC1s, and ILC3s also received signals from CXCR3 ligands (CXCL10, CXCL9, and PF4), which are mainly produced by myeloid cells and are known to play critical roles in lymphocyte infiltration (Fig. 7 C).

The analyses also identified tuft cells as an emerging communication hub in HFHS diet intestine and found that they exhibited dramatically increased immune-regulatory signal expression, such as *Vegfa*, *Efnb1*, *Lilrb4a*, *Pros1*, and *C3*. Tuft cells are also the major receivers for immune-regulatory signals from immune cells such as IL-4, IL-13, and CD83, and from structural cells, such as ephrin A1 (*Efna1*), ephrin A4 (*Efna4*), angiopoietin-2 (ANGPT2; *Angpt2*), and transthyretin (*Ttr*; Fig. 7 C and Fig. S5, A–C). Together, these data suggested significant communication shifts within the intestine interactome during HFHS diet and implicate new cell subsets in the potentiation of intestine inflammation and immune regulation in response to HFHS diet.

### A distinct HFHS diet-enriched intestine signalome uncovered

**Figure 8.**
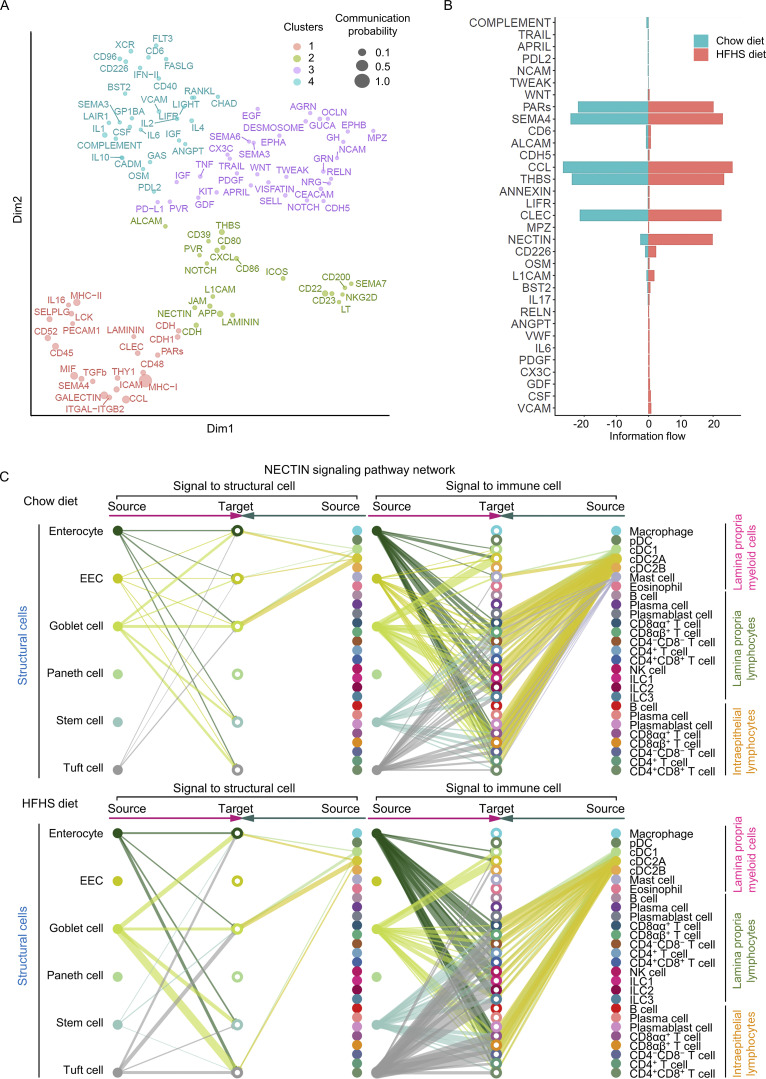
**Analysis of signaling pathways and information flow reveals a distinct intestine signalome in response to the HFHS diet. (A)** Jointly projecting and clustering signaling pathways from chow diet intestine and HFHS diet intestine onto shared 2D manifold according to functional similarity of the inferred networks. Each dot represents the communication network of one signaling pathway. Dot size is proportional to the communication probability. Different colors represent different groups of signaling pathways. IGF, insulin-like growth factor; GDF, growth differentiation factor; PDGF, platelet-derived growth factor; EGF, epidermal growth factor; EPHA, ephrin-A; EPHB, ephrin-B; APRIL, a proliferation-inducing ligand; SEMA, semaphorin; MPZ, myelin protein zero; GH, growth hormone; CEACAM, carcinoembryonic antigen-related cell adhesion molecules; RELN, reelin; TWEAK, TNF-related weak inducer of apoptosis; NRG, neuregulins; SELL, selection; LIFR, LIF receptor; ANGPT, angiopoietin; OSM, oncostatin M; ALCAM, activated leukocyte cell adhesion molecule; THBS, thrombospondin; JAM, junctional adhesion molecules; LT, lymphotoxin; PARs, protease-activated receptors; SELPLG, selectin P ligand; CLEC, C-type lectin-like receptor; MIF, macrophage migration inhibitory factor. **(B)** Significant signaling pathways were ranked based on differences in the overall information flow within the inferred networks between chow diet intestine and HFHS diet intestine. The overall information flow of a signaling network is calculated by summarizing all communication probabilities in that network. The top signaling pathways with P value < 1 × 10^−5^ were listed. Pathways colored red are enriched in HFHS diet intestine, and those colored blue were enriched in the chow diet intestine. A paired Wilcoxon test is utilized to determine whether there is significant difference between two datasets. **(C)** Hierarchical plot showing the inferred intercellular communication network of NECTIN signaling pathway at chow diet intestine and HFHS diet intestine, respectively. Circle color indicates each cell type and edge width are proportional to the communication probability.

To further understand how intestinal cells coordinate their functions, we used CellChat to conduct systems-level analysis through classifying conserved and context-specific signaling pathways (Jin et al., 2021). We projected signaling pathways onto a 2D manifold according to their functional similarity, which heavily weighs the similarities between sender and receiver cell group patterns, and revealed four groups of pathways (Fig. 8 A). Group #1 was dominated by signals produced by both epithelium cells and myeloid cells, such as TGFβ, PECAM, CLEC, and GALECTIN. Group #2 was dominated by co-stimulatory signals from myeloid cells and B cells, such as CD86, CD80, L1CAM, and ICOS. Group #3 mainly contained growth factor signals, such as IGF, GDF (growth differentiation factor), PDGF (platelet-derived growth factor), EGF, and GH (growth hormone). In addition, group #4 mainly consists of signals of immune response activation and regulation, such as IL-1, IL-2, IL-4, IL-6, IL-10, IFN-II, and RANKL. We then compared the information flow for each given signaling pathway by the sum of communication probability across chow and HFHS diet intestine and found that many group #1 pathways, including CD52, ICAM, MHC-I, LAMININ, PECAM, and GALECTIN maintain consistent in chow and HFHS diet intestine. They likely represented core signaling pathways essential for intestine function independent of the diet (Fig. S5 D). In contrast, 33 out of 101 pathways prominently changed information flow upon exposure to the HFHS diet. These consist mainly of signaling pathways from myeloid cells such as CCL, L1CAM, THBS, CLEC, NECTIN, and SEMA4. Presumably, these pathways are involved in inflammatory and intestinal homeostasis (Fig. S5 D). NECTIN pathways showed the most significant increase in information flow among these active pathways in the HFHS diet intestine (Fig. 8 B). Hierarchical plots indicated that the NECTIN signaling network in intestine is highly complex, with cDC2As and cDC1s as the sources of NECTIN ligand in immune cells, and with enterocytes, EECs, goblet cells, and tuft cells as the sources of the NECTIN ligand in structural cells. Meanwhile, the majority of NECTIN signal receivers are LPL-T cells, ILCs, and IEL-T cells, with minor receivers from structural cells (Fig. 8 C). In response to the HFHS diet, EECs lost their expression of NECTIN ligand and responsiveness while tuft cells dramatically increased their NECTIN ligand expression, driving an overall increase in NECTIN communication (Fig. 8 C). The interactions between nectin-2 on APCs and its ligand CD226 on NK cells and T cells have been reported to play an important role in T cells and NK cells activation (Huang et al., 2020; Tahara-Hanaoka et al., 2004; Yeo et al., 2021). Interestingly, CD226, grouped together with signaling pathways involving immune cell activation in group #4 (Fig. 8 A), is also among the most significantly upregulated signaling pathways in HFHS diet intestine (Fig. 8 B and Fig. S5 D). This suggests that the NECTIN–CD226 signaling pathway plays an important role in the activation of LPL-T, ILCs, and IEL-T cells, and the intestinal homeostasis in response to the HFHS diet.

**Figure 9.**
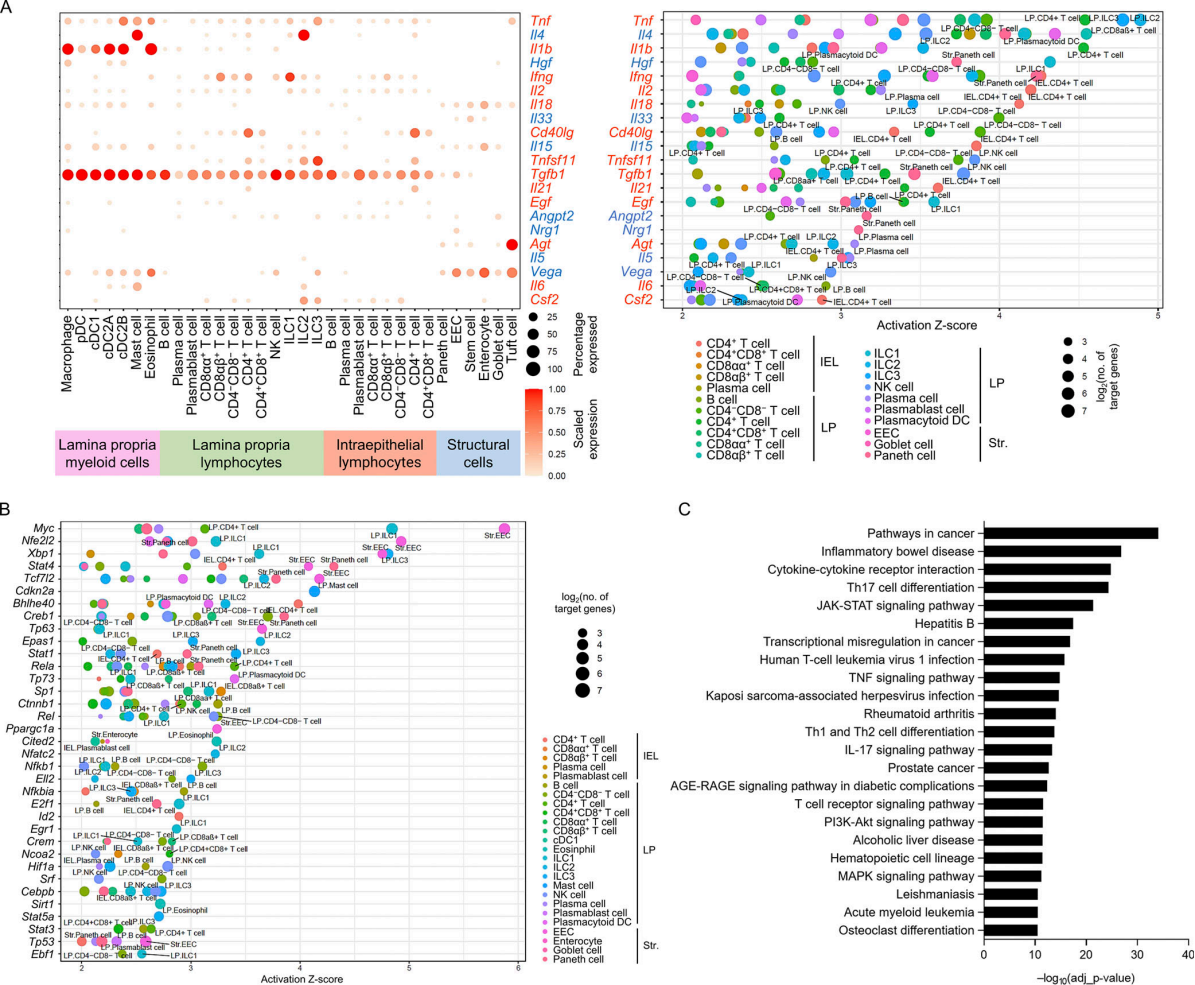
**Analysis of upstream regulators uncovers a distinct intestine signalome in response to the HFHS diet. (A and B)** IPA of HFHS immune and non-immune populations showing common upstream regulators. Terms were considered common if implicated in 10 or more cell types from a lineage (Tables S8, S9, and S10). Terms were considered statistically significant if the activation z-score >2. **(A)** Dot plots showing expression of common secreted upstream regulators from HFHS diet intestinal cells (left) and the putative regulated cell types (right) as suggested by IPA. Color saturation in dot indicates the strength of expression in specific cell type, whereas dot size reflects the percentage of cells in each population expressing the gene (left). The color indicates the implicated cell type, whereas dot size reflects the number of genes downstream of the suggested secreted upstream regulator. Red-highlighted upstream regulators denote those that have been associated with obesity or metabolic dysfunction. Blue-highlighted upstream regulators denote those that have been associated with immune homeostasis or obesity resistance. **(B)** Dot plot showing common transcriptional signaling upstream regulators. The color indicates the implicated cell type, whereas dot size reflects the number of genes downstream of the suggested signaling upstream regulator. IEL, intraepithelial lymphocyte; LP, lamina propria; Str., structural cells. **(C)** Top differentially regulated signaling pathways in response to HFHS diet suggested in g:GOSt functional enrichment analysis of common upstream regulators. Upstream regulators were considered common if implicated in 10 or more cell types from a lineage. Terms with −log_10_ (adjusted P value) >10 were shown.

To further explore whether predicted interacting cell types influence transcriptional changes during the HFHS diet challenge, we performed IPA on the DEGs between all chow diet and HFHS diet cell populations (Tables S8 and S9). Our analysis identified ∼269 genes implicated as shared upstream regulators in ≥10 cell types (Table S10). The analysis indicated that many signaling pathways identified utilizing CellPhoneDB and CellChat served as upstream regulators of gene expression in intestinal cells in response to a HFHS diet (Fig. 9 A). Many of the common secreted upstream regulators we identified, such as TNF, IL-1β, IFN-γ, IL-18, CD40L, IL-6, EGF, TGFβ, and IL-21, were previously reported to be associated with obesity and metabolic dysfunction (Table S11). Moreover, our analysis suggested that many previously uncharacterized intestinal immune cells, including CD4^+^ IEL-T cells, ILC1s, ILC2s, and CD4^+^ LPL-T cells, expressed high levels of the downstream genes of these key inflammatory pathways associated with obesity, which may contribute to their proliferation and infiltration, in accordance with changes in cell proportion and ligand–receptor interaction analysis (Fig. 7 C and Fig. 9 A).

Our analysis also identified several secreted upstream regulators that have not yet been associated with intestinal inflammation but have been suggested to play a role in the development of metabolic dysfunction (GM-CSF, TNFSF11, IL-2, and Angiotensinogen [Agt]), suggesting that these signals may exert their systemic effects through targeting intestinal immune cells (Table S11). Furthermore, our results also revealed several common upstream regulators associated with immune regulation and resistance to obesity (IL-4, IL-5, IL-33, CD15, HGF, ANGPT2, VEGFA, and neuropilin 1 [Nrp1]), supporting a critical role of intestinal homeostasis in systemic pathogenesis of obesity (Table S11). Many previously uncharacterized structural cells including tuft cells, EECs, stem cells, and enterocytes highly expressed the upstream ligands of many of these immunoregulatory pathways in the obesifying diet, indicating their potential roles in regulating intestinal immune homeostasis (Fig. 9 A). Analysis of non-secreted upstream regulators indicated that EECs, Paneth cells, ILC1s, ILC2s, and CD4^+^ IEL-T cells expressed the highest levels of the genes downstream of the inflammatory signaling regulators (such as *Myc*, *Nfe2l2*, *Xbp1*, *Stat4*, and *Tcf7l2*), indicating those intestinal cells may play an important role contributing to obesity-associated inflammation and metabolic dysfunction (Fig. 9 B). Together, these results further supported the identification of pro-inflammatory signal producers in the ligand-receptor interactome (Fig. S5, E–H).

Functional enrichment analysis of the common upstream regulators suggested a number of significant pathways that are transcriptionally regulated in HFHS diet intestine. These involved several pro-inflammatory signaling pathways, including IL-17, JAK-STAT, TNF, PI3K-Akt, AGE-RAGE, and MAPK (Fig. 9 C). Interestingly, signaling pathways in cancer are at the top of the list of most differentially regulated signaling pathways in response to HFHS diet. It is known that dietary fat intake is associated with an elevated risk of developing colorectal cancer (Keum and Giovannucci, 2019). Our results suggest that upstream regulators such as MYC, TP53, FOXO1, H1F1A, and FOS might contribute to an increased risk of cancer development in the intestine in response to an HFHS diet (Table S12).

We followed up on the XCR1–XCL1 signaling pathway, which is known to be critical for cDC1 recruitment. To study XCR1–XCL1 signaling pathway in vivo, we utilized anti-XCL1 antibodies i.p. injection to block the XCR1–XCL1 signaling pathway in mice fed on a HFHS diet (Fig. S5 I). The immune cells of the epithelium layer and lamina propria layer were isolated from both the XCL1 signaling blockade group and the control group, and then analyzed by flow cytometry for major immune cell populations (Fig. S5 I). The results show that abrogation of cDC1s increased the CD8αα^+^ IEL-T cells in the epithelium layer and CD4^+^ LPL-T cells in the lamina propria layer in the mouse intestine (Fig. S5, J and K). These results indicate that XCL1–XCR1 signaling pathway is involved in the regulation of inflammatory responses and intestinal homeostasis upon HFHS diet feeding. Significant CD4^+^ LPL-T cell infiltration was observed in the intestine upon HFHS diet feeding (Fig. 1, E–G), and cDC1s were the major receiver and the sender of a variety of immune-regulatory signals, and they appeared to play a central role in the responses to a HFHS diet (Fig. 7, A–C). These results suggest that cDC1s may play a regulatory role in intestinal homeostasis and modulate the CD4^+^ LPL-T cells and CD8αα^+^ IEL-T cells activation upon HFHS diet feeding. Furthermore, to study the impact of cDC1 abrogation on IECs upon HFHS diet feeding, we also sorted non-hematopoietic CD45.2^−^ live cells from the epithelium layer of the mice from XCL1–XCR1 signaling blockade group and the control group, and then profiled them using RT-QPCR (Table S13). We examined the gene expression of transporters for key nutrient families, and found that amino acid transporters (*Slc7a8*, *Slc7a9*), and peptide transporters (*Slc15a1*, *Slc15a2*) were increased in the XCL1–XCR1 signaling blockade group, whereas the expression of the carbohydrate transporters (*Slc2a2*, *Slc2a5*) decreased. The genes involved in fat and cholesterol absorption (*Apob*, *Apoa1*, *Npc1l1*) remain unchanged (Fig. S5 L). Taken together, these results indicate that cDC1s also play an important role in regulating IEC functions, further supporting their role as a central communication hub in the HFHS diet intestine.

We also studied the NECTIN pathway, the most significantly increased pathway in information flow in response to the HFHS diet, in vivo. We utilized anti-CD226 antibodies i.p. injection to block the NECTIN–CD226 signaling pathway in mice fed on a HFHS diet (Fig. S5 M). The immune cells of two major intestinal compartments, the epithelium layer and lamina propria layer, were isolated from both the NECTIN signaling blockade group and the control group, and then analyzed by flow cytometry for major immune cell populations (Fig. S5 M). The results show that there was no significant change in intraepithelial immune cell populations (Fig. S5 N). However, dramatic decreases of CD4^+^ LPL-T cells, CD4^−^CD8^−^ LPL-T cells and NK cell/ILC1s were observed in the intestine of mice with the CD226 signaling blockade (Fig. S5 O). In contrast, the proportions of myeloid cells, including cDC2As, cDC2Bs, and macrophages, increased (Fig. S5 O). These results indicate that NECTIN–CD226 signaling pathway is involved in inflammatory responses and intestinal homeostasis. Moreover, the NECTIN pathways showed the most significant increases in information flow among active pathways in the HFHS diet intestine (Fig. 8 B), and the majority of NECTIN signal receivers are T cells and ILCs (Fig. 8 C). Taken together, these results suggest that the NECTIN–CD226 signaling pathway plays an important role in the accumulation of CD4^+^ LPL-T cells, CD4^−^CD8^−^ LPL-T cells and ILCs, promoting intestinal inflammation in the HFHS diet feeding mice.

Combined, these data suggest that a complex network of inflammatory and immunomodulatory signaling pathways in both immune cells and structural cells regulates the homeostasis in the intestine during HFHS diet-induced obesity.

## Discussion

We performed scRNA-seq on a total of 70,830 cells from multiple sorted atlases including intraepithelial CD45^+^ cells, lamina propria CD45^+^ cells, and epithelium CD45^−^ cells from mice on chow and HFHS diets. This led to the validation of 32 out of 32 previously identified populations, as well as the identification of an additional 20 distinct cell clusters, including previously uncharacterized populations of CD8αα^+^ IEL-T cell, CD8αβ^+^ IEL-T cells, CD4^+^ IEL-T cells, CD4^+^ LPL-T cells, cDCs, and enterocytes. Using flow cytometry, we further validated the presence of each immune cell population in the chow diet and HFHS diet intestines using an independent cohort of mice. Finally, we utilized the single-cell ligand–receptor analysis to profile both the chow diet and the HFHS diet intestine interactomes in a cell type-specific way, establishing a signalome across intraepithelial immune cells, lamina propria immune cells and epithelial structural cells in the intestine.

**Figure 10.**
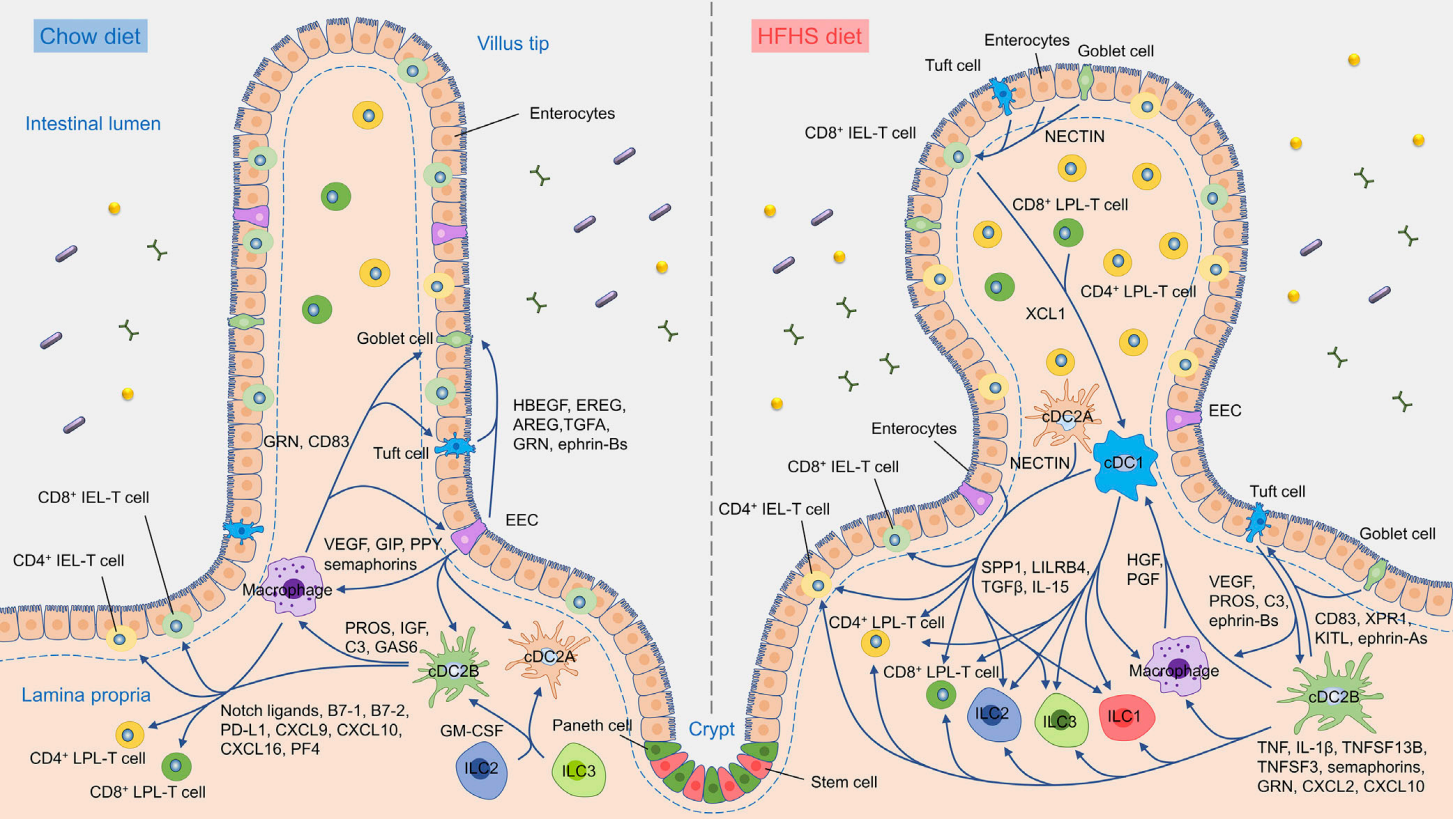
**Graphical summary of cell–cell interactions in healthy and HFHS diet intestine.** In healthy intestine, cDC2Bs and macrophages play as central communication hubs, regulating IEL-T cells and LPL-T cells via Notch ligands, B7-1, B7-2, PD-L1, CXCL9, CXCL10, CXCL16, and PF, and regulating EECs, tuft cells, and goblet cells via GRN and CD83. cDC2Bs are also the major producers of immunoregulatory signals such as PROS, IGF, C3, and GAS6. Among structural cells, EECs play as a central communication hub and interact with macrophages, cDC2Bs, and cDC2As via VEGF, GIP, PPY, and semaphorins. EECs and tuft cells may support goblet cells via production of HBEGF, EREG, AREG, TGFA, GRN, and ephrin-Bs. ILC2 and ILC3 also support cDCs through GM-CSF production. During HFHS diet-induced obesity, EECs decrease their cell–cell interactions while tuft cells become the major signal source among structural cells. Tuft cells may regulate with macrophages and cDC2Bs via VEGF, PROS, C3, and ephrin-Bs, and receive signals from goblet cell and cDC2Bs via CD83, XPR1, KITL, and ephrin-As. Notably, tuft cells, goblet cells, and enterocytes increase their interactions with CD8^+^ IEL-T cells via NECTIN signaling in HFHS diet intestine. Increased XCL1 production of CD8^+^ IEL-T cells could further recruit more cDC1s in the lamina propria. As a communication hub in HFHS diet intestine, cDC1 produce immunoregulatory signals such as SPP1, LILRB4, TGFβ, and IL-15, and received HGF and PGF signals from macrophage and cDC2B. cDC1 and cDC2A could also interact with IEL-T cells and LPL-T cells via nection-2 signaling. In contrast, cDC2B produce signals such TNF, IL-1β, TNFSF13B, TNFSF3, semaphorins, GRN, CXCL2, CXCL10, and potentiate inflammation in the HFHS diet intestine. EECs, enteroendocrine cells; GRN, granulin; PROS, protein S; IGF, insulin-like growth factor; GAS6, growth arrest-specific 6; VEGF, vascular endothelial growth factor; GIP, gastric inhibitory polypeptide; PPY, pancreatic polypeptide; HBEGF, heparin binding EGF-like growth factor; EREG, epiregulin; AREG, amphiregulin; XPR1, xenotropic and polytropic retrovirus receptor 1; KITL, KIT ligand; SPP1, secreted phosphoprotein 1; HGF, hepatocyte growth factor; PGF, placental growth factor.

Our studies identified unique immune cell populations that accumulate in response to the HFHS diet and the associations between them. For example, Treg cell proportions were dramatically increased in the HFHS diet, as were CD4^+^ IEL-T cells and CD4^+^ LPL-T cells, and cDC1s, which are critical for Treg induction. Moreover, memory-like CD8αα^+^ IEL-T cells as well as memory-like CD8αβ^+^ IEL-T cells accumulated in HFHS diet intestine, expressed high levels of XCL1, and are likely involved in the recruitment of cDC1s via XCL1–XCR1 signaling axis in response to the diet. This mechanism is very similar to the cDC1 recruitment and immune tolerance induction mechanism discovered in the tumor microenvironment (Böttcher et al., 2018). Although the percentage of Treg cells increased in CD4^+^ T cell populations (Fig. 1 D), the RT-QPCR analysis and absolute cell counting indicate increased Th1 and Th17 responses in the lamina propria due to increased CD4^+^ T cell infiltration (Fig. S3, F and G). Analysis of the intestine interactome suggested that CD4^+^ T cells are one of the major producers of many strong pro-inflammatory signals (Fig. 7 C). Furthermore, IPA analysis indicated that CD4^+^ T cells expressed high levels of the downstream genes of the key inflammatory pathways associated with obesity (Fig. 9, A and B). These results suggest that CD4^+^ T cells also play a pro-inflammatory role in response to the HFHS dietary challenge. Notably, cDC1s were the major receiver and the sender of a variety of immune-regulatory signals, and they appeared to play a central role in the responses to a HFHS diet. Moreover, the abrogation of cDC1s significantly increased the CD4^+^ LPL-T cell and ILCs infiltration in the intestine upon HFHS diet feeding. These results suggested a previously unrecognized role of the CD8^+^ IEL-T cell–cDC1–CD4^+^ T cell axis in the balance of intestinal homeostasis (Fig. 10).

Our studies also spatially reconstituted the stem cell to enterocyte development trajectory in the intestine villus. We found that the transporters of the key nutrient families (amino acid, peptide, lipid, and carbohydrate) have their own expression domains along the developmental timeline zonation from stem cell to villus tip. We also found that IECs in the intestine undergo unique adaptions in response to the HFHS diet, especially the expression of distinct nutrient transporters. Moreover, it has long been known that the essence of immune responses, whether to infection, in autoimmunity, or to cancer, is orchestrated by the principal cell populations of the immune system, that is, lymphocytes and myeloid cells. However, our present results indicate that structural cell types also dictate the immune responses. For example, in response to HFHS diet feeding, EECs reduced expression of the NECTIN signal, which is actively involved in LPL-T cell and IEL-T cell activity in healthy intestine. Furthermore, IPA analysis also showed that EECs expressed the highest levels of genes responsive to the inflammatory intestinal microenvironment. Intestine interactome analyses also indicated that EECs act as a central communication hub among structural cells and largely lose their status to tuft cells in response to the HFHS diet (Fig. 10). These findings highlight the study of IEC populations in regulating the immune homeostasis and systemic metabolism.

A limitation of our study is that we did not incorporate the effects of the gut microbiome in our analysis. However, we did observe that the expression of genes involved in microbiota–host interactions was significantly affected by Western diet (Fig. S4, H and I).

In summary, our study provides a comprehensive landscape of intestinal immune cell and structural cell populations on a chow diet and in response to a HFHS diet. It also identified cell type-specific transcriptional changes and communication networks that underlie intestinal homeostasis on the two diets. Our study filled a knowledge gap in analyzing the different layers of intestinal cells and interaction networks across immune cells and structural cells. The study revealed many previously uncharacterized intestinal immune cells and their potential roles in inflammatory pathways, and identified many upstream regulators in obesity-associated inflammation, suggesting that they may exert their systemic effects through targeting specific intestinal cells.

## Materials and methods

### Mice

C57BL/6J (B6) mice were purchased from the Jackson Laboratory. 8-wk-old female mice were used for all experiments unless otherwise indicated. All experiments were repeated at least three times unless specifically mentioned. Replicates of each individual experiment are detailed in the figure legends. All animals were maintained at the University of California, Los Angeles (UCLA) animal facilities and all experiments were approved by the Institutional Animal Care and Use Committee of UCLA (ARC-92-169). Mice were maintained on a 12-h light/dark cycle from 6 am to 6 pm at ambient temperature (∼72°F degree) with controlled humidity (∼45%) in specific pathogen-free conditions. Animals were randomly assigned to each treatment group and experiments were performed under standard laboratory procedures of randomization and blinding. For HFHS diet mice models, 8-wk-old mice were fed with either a chow diet (Cat# 2916; Teklad) or a HFHS diet (Cat# D12266B; Research Diets).

### Isolation of mouse intraepithelial immune cells, lamina propria immune cells, and IECs

Mice were euthanized using isoflurane (VETONE) and secondary cervical dislocation. The intestine cell isolation protocols were optimized by our lab following the guidelines (Lefrancois and Lycke, 2001; Qiu and Sheridan, 2018; Sydora et al., 1996). The intestines were next maintained in a moistened state, and the Peyer’s patches and the remaining mesentery/fat were excised. The small intestine from ileum to jejunum was collected and gently flushed with a syringe with ice-cold 1× Hanks’ balanced salt solution (HBSS) media (without Ca^2+^ and Mg^2+^; Cat# H4641; Sigma-Aldrich) to remove the feces. The intestine was cut open and dissected into 1-cm segments, which were then transferred into a 50 ml tube and gently shaken to let the tissue stretch. They were then washed twice with ice-cold 1 × HBSS and the extra HBSS was removed using a pipet. 20 ml pre-digestion solution (1× HBSS with 5% FBS, 5 mM EDTA, and 1 mM DTT) was added and incubated for 20 min at 37°C with 250 rpm rotation in an incubator. The 1× HBSS containing the epithelial cell layer was collected on ice after the vortex and passed through a 100-μm cell strainer.

For intraepithelial immune cell isolation, the 1× HBSS containing the epithelial cell layer was centrifuged at 2,000 rpm for 5 min. The cell pellet was resuspended in 20 ml 40% percoll (Cat# P4937; Sigma-Aldrich) and 20 ml 70% percoll added to the bottom of each tube, spun at 2,000 rpm for 20 min (accelerate at 6 and brake at 2) at room temperature. The middle layer of cells was collected, washed, spun down with 1× PBS, and resuspended in RPMI 1640 culture media (Cat# 10-040-CV; Corning Cellgro; supplemented with 10% FBS (Cat# MT35015CV; Corning), 1% penicillin-streptomycin-glutamine (Cat# 10378016; Gibco), 1% MEM Non-Essential Amino Acids Solution (Cat# 11140050; Gibco), 1% HEPES [Cat# 15630056; Gibco], and 1% sodium pyruvate [Cat# 11360070; Gibco]) for further FACS or flow cytometry analysis. For intraepithelial immune cell scRNA-seq, single cell suspensions were sorted using a FACSAria III flow cytometer to purify live hematopoietic cells (gated as DAPI^−^CD45.2^+^ cells).

For IEC isolation, the epithelial cell layer in the 1× HBSS was spun at 2,000 rpm for 5 min. The cell pellet was resuspended in RPMI 1640 culture media for further FACS. For epithelial structural cell scRNA-seq, single cell suspensions were sorted using a FACSAria III flow cytometer to purify live non-hematopoietic cells (gated as DAPI^^−^^CD45.2^−^ cells).

For lamina propria immune cell isolation, the epithelial cell layer was removed and the remaining intestine tissues were washed twice with RPMI 1640 culture media. The extra media was removed and 10 ml RPMI 1640 culture media with 1 mg/ml Collagenase type I (Cat# 17100017; Thermo Fisher Scientific) was added. The sample was incubated for 20 min at 37°C with 250 rpm rotation and vigorously vortexed to allow the tissue to fully dissolve before passing through 100-μm cell strainer. Media containing the lamina propria cells was centrifuged at 2,000 rpm for 5 min and the cell pellet was resuspended in 20 ml 40% percoll. 20 ml 70% percoll was added to the bottom of each tube and spun at 2,000 rpm for 20 min (accelerate at 6 and brake at 2) at room temperature. The middle layer of cells was collected, washed, spun down with 1× PBS, and resuspended in RPMI 1640 culture media for further FACS or flow cytometry analysis. For lamina propria immune cell scRNA-seq, single cell suspensions were sorted using a FACSAria III flow cytometer to purify live hematopoietic cells (gated as DAPI^−^CD45.2^+^ cells).

### mIHC staining

Small intestines from the ileum to jejunum were collected from experimental mice at the termination of an experiment and kept moistened. They were gently flushed with ice-cold 1× HBSS media to remove the feces with a syringe and cut into 1-cm-long segments. The parts of ileum 2 cm away from the cecum were collected for further mIHC analysis.

Intestine sections were stained with the manual Opal 7-Color IHC Kit (Cat# NEL811001KT; Akoya Biosciences) with modification. Intestinal sections were prepared with a formalin-fixed, paraffin-embedded (FFPE) technique. The slides were dewaxed with xylene (3 × 10 min) and rehydrated through a graded series of ethanol solutions: (100% 1 × 5 min; 95% 1 × 5 min; and 70% 1 × 2 min) and washed in distilled water (1 × 2 min) and TBST (1 × 2 min). Then slides were then placed in a plastic jar with AR buffer and boiled by microwave for 45 s at 100% power and an additional 15 min at 20% power. Then slides were washed in distilled water (1 × 2 min) and TBST (1 × 2 min). The tissue sections were covered with blocking buffer (PerkinElmer antibody diluent buffer, Cat# ARD1001EA) and incubated in a humidified chamber for 10 min at room temperature. Then primary antibodies were applied overnight or at room temperature for 1 h according to antibody sensitivity. After incubation, slides were washed with TBST three times and incubated with secondary antibodies for 30 min at room temperature. Slides were washed three times with TBST and incubated with opal Fluorophore Working Solution (1:50 dilution) for 10 min to amplify the signals. After signal amplification, slides were washed three times with TBST, boiled with a microwave in AR buffer and washed with distilled water and TBST to strip the antibody complex. The steps of blocking, first antibody and secondary antibody were repeated for each antibody. After the incubation of all antibodies, slides were incubated with DAPI (1:2,000 dilution) and mounted. 32 sections from 16 mice in each group were studied by mIHC. An average of the two sections from the same mouse was used for the quantification of the sample.

For IHC of intestine tissues, FFPE intestine samples were stained with anti-CD3 (Cat# A0452; Agilent), anti-CD8α (Cat# 14-0808; eBioscience), anti-CD4 (Cat# AB183685; Abcam), and anti-Ki67 (Cat# 12202; Cell Signaling) as primary antibodies. EnVision + System-HRP, Labelled Polymer (goat anti-mouse; Cat# K4001; Agilent), EnVision + System-HRP, Labelled Polymer (goat anti-rabbit; Cat# K4003; Agilent), rabbit anti-rat IgG antibody (Cat# BA-4001; CiteAb) were utilized for secondary antibody staining. All fluorescently labeled slides were scanned on the Vectra Polaris (Akoya Biosciences) at 40× magnification using appropriate exposure times. The data from the multispectral camera were analyzed by the imaging InForm software (Akoya Biosciences) and Phenochart software (Akoya Biosciences).

### H&E staining

Small intestine segments were prepared as above (mIHC) and subjected to a FFPE technique. Slides were prepared in 4-μm sections and dewaxed with xylene (Cat# X5-4; Thermo Fisher Scientific; 3 × 10 min), rehydrated through a graded series of ethanol solutions (100% 3 × 10 min; 95% 2 × 5 min; and 70% 1 × 10 min), and washed in distilled water (1 × 5 min). Samples were stained with hematoxylin (1 × 10 min; Cat# SH26-500D; Thermo Fisher Scientific) and washed in distilled water again (1 × 10 min). Samples were then dipped into acid alcohol (two to five times) before being washed in distilled water (1 × 10 min). They were then dipped into sodium bicarbonate solution (Cat# BP328-1; Thermo Fisher Scientific) 20 times. Samples were then washed in water (1 × 10 min), and dehydrated through a graded series of ethanol solutions with eosin staining in the order: 70% alcohol (20 dips), eosin (1 × 3 min), 95% alcohol (2 × 20 dips), 100% alcohol (3 × 5 min). Lastly, the slides went through xylene (4 × 5 min) and slips were covered. All slides were scanned on the Aperio AT scanning system (Leica) using appropriate exposure times.

### In vivo modeling of antibody blockade in HFHS diet fed mice

In some experiments, 8-wk-old male mice received i.p. injection of anti-XCL1 antibody (25 µg per mouse; once per week; Cat# AF486; R&D Systems) to block XCL1 activity 1 d before HFHS diet feeding. IgG isotype control (25 µg per mouse; once per week; Cat# AB-108-C; R&D Systems) was utilized in the control group. In some experiments, 8-wk-old male mice received i.p. injection of anti-CD226 antibody (25 µg per mouse; once per week; Cat# 132010; Biolegend) to block XCL1 activity 1 d before HFHS diet feeding. IgG isotype control (25 µg per mouse; once per week; Cat# 400544; Biolegend) was utilized in the control group. Mice were fed a HFHS diet (Cat# D12266B; Research Diets). Following euthanasia, intraepithelial immune cells, and lamina propria immune cells were isolated for flow cytometry analysis. IECs were isolated and sorted using a FACSAria III flow cytometer (gated as DAPI-CD45.2^−^ cells) for QPCR analysis.

### Real-time quantitative PCR (RT-QPCR)

Total RNA was extracted from cells using TRIzol reagent (15596018; Invitrogen; Thermo Fisher Scientific) following the manufacturer’s instructions. iScript cDNA Synthesis Kit (Bio-Rad) was used for reverse transcription. QPCR was performed using the PowerUp SYBR Green Master Mix (Applied Biosystems) and the iCycler Real-time PCR Detection System (Bio-Rad) according to the manufacturer’s instructions. Housekeeping gene Ube2d2 was used as an internal control for mice cells. The relative expression of a target gene was calculated using the ΔΔCT method. All sequences of primers used in this study are listed in Table S13.

### Flow cytometry

Flow cytometry was used to analyze surface marker and intracellular effector molecule expression in immune cells. Fluorochrome-conjugated monoclonal antibodies specific for mouse CD45.2 (clone 104; Cat# 109830), CD11b (clone M1/70; Cat# 101205), F4/80 (clone BM8; Cat# 123126), CD11c (clone N418; Cat# 117324), Ly-6G (clone 1A8; Cat# 127612), CD19 (clone 6D5; Cat# 115507), NK1.1 (clone PK136; Cat# 108710), I-A/I-E (clone M5/114.15.2; Cat# 107623), TCRβ (clone H57-597; Cat# 109220), TCRγ/δ (clone GL3), CD4 (clone RM4-5; Cat# 100531), CD8α (clone 53-6.7; Cat# 100732), CD8β (clone YTS156.7.7; Cat# 126605), Ki-67 (clone 16A8; Cat# 652403), SIRPα (clone P84; Cat# 144007), CD103 (clone 2E7; Cat# 121405), CD3 (clone 17A2; Cat# 100221), XCR1 (clone ZET; Cat# 148205), CD138 (clone 281-2; Cat# 142505), CD38 (clone 90; Cat# 102717), PDCA-1 (clone 927; Cat# 127015), IFNγ (clone XMG1.2; Cat# 505849), IL17A (clone TC11-18H10.1; Cat# 506915) and Granzyme B (clone QA16A02; Cat# 372204) were purchased from Biolegend. Mouse Fc Block (anti-mouse CD16/32; clone 2.4G2; Cat# 553142) was purchased from BD Biosciences. FVD eFluor 506 (Cat# 65-0866-14) was purchased from Thermo Fisher Scientific to exclude dead cells in flow cytometry. Foxp3 (clone FJK-16 s; Cat# 11-5733-82) was purchased from eBiosciences. DAPI (1:1,000 dilution) was utilized to exclude dead cells in FACS.

To study cell surface marker expression, cells were stained with FVD followed by Fc blocking and surface marker staining, following standard procedures described previously (Wang et al., 2021). For T cell intracellular cytotoxicity molecule and transcription factor production, intracellular staining of Granzyme B and Ki-67 was performed directly using the eBioscience Foxp3/Transcription Factor Staining Buffer Set (Cat# 00-5523-00; Invitrogen) following the manufacturer’s instructions. These cells were co-stained with surface markers to identify CD8αα^+^ IEL-T cells and CD8αβ^+^ IEL-T cells (gated as FVD^−^CD45.2^+^CD3^+^CD4^−^CD8α^+^CD8β^−^ cells and FVD^−^CD45.2^+^CD3^+^CD4^−^CD8α^+^CD8β^+^ cells in vivo). For T cell intracellular cytokine production, CD4^+^ T cells were stimulated with PMA (VWR; 50 ng/ml) and Ionomycin (VWR; 500 ng/ml) in the presence of GolgiStop (BD Biosciences; 4 μl per 6 ml culture media) for 4 h. At the end of the culture, cells were collected and intracellular cytokine (i.e., IFNγ and IL17A) staining was performed using the eBioscience Foxp3/Transcription Factor Staining Buffer Set (Cat# 00-5523-00; Thermo Fisher Scientific) and following the manufacturer’s instructions. These cells were co-stained with surface markers to identify CD4^+^ T cells (gated as FVD^−^CD45.2^+^CD3^+^CD8α^−^CD4^+^ cells in vivo). Stained cells were analyzed using a MACSQuant Analyzer 10 flow cytometer (Miltenyi Biotec). Data were analyzed using FlowJo software (BD Biosciences).

### FACS (fluorescence-activated cell sorting)

Single cell suspensions were harvested from intestines of chow diet-fed and HFHS diet-fed mice and then pooled for further sorting (10 mice were combined for each group). To isolate different cell populations, cells were treated with Fc blocking followed by surface marker staining, following a standard procedure (Wang et al., 2021). DAPI (1:1,000 dilution) was added to single cell suspensions 5 min before sorting to exclude dead cells. Cells were then sorted using a FACSAria III flow cytometer to purify intraepithelial and lamina propria hematopoietic cells (gated as DAPI^−^CD45.2^+^ cells) and epithelial structural cells (gated as DAPI^−^CD45.2^−^ cells). Sorted live cells were counted and immediately utilized for library construction and sequencing. To reduce the potential batch effects in experimental design and handling, 10 mice were sacrificed at the same time for each sample, and all samples were sent to QC and library construction at the same time.

### Library preparation, sequencing, and alignment

Cells were stained with trypan blue (Cat# T10282; Thermo Fisher Scientific) and counted using a Cell Countess II automated cell counter (Thermo Fisher Scientific). Libraries were constructed using a Chromium Single Cell 3′ library (10× Genomics) & Gel Bead Kit V2 (10× Genomics, Cat# PN-120237) according to the manufacturer’s instructions. Libraries were sequenced using the NovaSeq 6000 S2 Reagent Kit (Illumina) to a depth around 2 billion reads per library using 2 × 50 read length. Data analysis was performed using a Cellranger Software Suite (10× Genomics). Individual samples were extracted from the sequencer and used as inputs for the Cellranger pipeline to generate the merged digital expression matrix using Cellranger aggregation. The raw data of all samples was output by Cellranger at the same time after the aggregation process. We followed the effective design of single-cell gene expression studies to reduce the potential batch effects (Luecken and Theis, 2019).

### Cell clustering and annotation

The merged digital expression matrix generated by Cellranger was analyzed using Seurat (v.4.0.0) following the guidelines. Seurat is an R package developed in 2017 by the Satija lab for single cell RNA sequencing (Butler et al., 2018). We also used the recommended methods for batch integration (Tran et al., 2020). Specifically, cells were first filtered to have at least 100 unique molecular identifiers, at least 100 genes, at most 10% mitochondrial gene expression for intraepithelial and lamina propria immune cells, and at most 50% for IECs. The gene counts for each cell were divided by the total gene counts for the cell and multiplied by a scale factor of 10,000, then normalized using the Seurat function NormalizeData through natural-log transformation. Normalization steps were employed to eliminate technical noise or bias so that observed variance in gene expression variance primarily reflects true biological variance. This normalization targets variance from sequencing (library preparation, high dropout event, amplification bias caused by gene length GC content, etc.; Jia et al., 2017).

Next, variable genes were found using the Seurat function FindVariableGenes. The ScaleData function was used to regress out the sequencing depth for each cell. Variable genes that had been previously identified were used in principal component analysis (PCA) to reduce the dimensions of the data. Following this, 50 principal components were used in UMAP to further reduce the dimensions to 2. The same 50 principal components were also used to group the cells into different clusters by the Seurat function FindClusters. Next, cluster marker genes were found for each cluster using the FindAllMarkers function. Cell types were manually annotated based on the cluster markers. Module scores were calculated using the AddModuleScore function for certain functions and metabolic processes. To calculate the total sample composition based on cell types, the number of cells for each cell type was counted. The counts were then divided by the total number of cells and scaled to 100% for each cell type. For the proportion of cell subclusters, the number of cells for each cell subcluster was counted and then divided by the total number of that cell type before scaling to 100%. For potential upstream regulator analyses, differential expression analysis was carried out between cell subclusters. Then IPA was applied to the DEGs to determine the potential upstream regulators driving the differential expression.

### CytoTRACE analysis

The gene expression matrix for certain cell types was extracted with single cell IDs and the gene names. The databases were uploaded to the CytoTRACE website (https://cytotrace.stanford.edu/) to predict differentiation states. CytoTRACE score of each cell was calculated and integrated back into the scRNA-seq database and plotted on the UMAP afterward. 3D graphics were generated using the CytoTRACE website features. 2D graphics were generated utilizing the FeaturePlot function.

### Pseudotime trajectory

Pseudotime trajectories were analyzed using the R package Monocle3 (v.2.18.0; Trapnell et al., 2014). After clustering analysis, the data dimensionality of the intended cell types were reduced using UMAP. Monocle was utilized to learn trajectory through cluster_cells function by the default method. Next, we fit a principal graph within each partition using the learn_graph function and trajectories with numerous branches were reconstructed. After specifying the root nodes of the intended trajectory, we used the order_cells function to calculate where each cell falls in pseudotime of the biological process. Finally, the plot_cells function was utilized to show the trajectory graph in UMAP and to color cells by pseudotime. Heatmaps showing the bifurcation expression patterns were generated using function plot_genes_branched_heatmap with DEGs with the FDR adjusted P value <1e−50.

### RNA-velocity analysis

To estimate the RNA velocity of samples, velocyto (La Manno et al., 2018) was used to distinguish unspliced and spliced messenger RNAs in each cell and to recover the directed dynamic information by leveraging mRNA-splicing information. Specifically, .LOOM files with spliced/unspliced expression matrices were generated for each sample. The Velocyto.R-package (v0.6) was utilized for further analysis. After loading .LOOM files information through ReadVelocity function, databases were merged and RunVelocity function was performed to obtain the velocity vectors. Finally, the velocities were projected into a lower-dimensional embedding using the velocity_graph function and visualized on the UMAP embedding in each intended cell cluster using the show.velocity.on.embedding.cor function. All velocyto functions were used with default parameters.

### Cell–cell ligand–receptor interaction

CellphoneDB (v.2.1.7), CellTalkDB (v. 0.0.1.6), and CellChat (v.1.1.3) were utilized for ligand–receptor analysis. The raw gene expression counts and cell type annotation for each single cell were analyzed with the CellphoneDB database to determine the potential ligand–receptor pairs and filtered with a threshold of *P*-value below 0.05. Four groups of major cell types: intraepithelial lymphocytes, lamina propria lymphocytes, lamina propria myeloid cells (accumulated with additional cohort), and structural cells from intestine epithelium were merged and two runs were performed on chow diet intestinal cells and HFHS diet intestinal cells, respectively. The number of interactions between each pair of cell types was plotted based on source and receptor using heatmap and circle interactome. Selected ligand–receptor pairs were plotted in dot plot to indicate expression patterns across all cell types. All ligand–receptor pairs exhibited were manually verified via CellTalkDB, a manually curated database of literature-supported ligand–receptor interactions (http://tcm.zju.edu.cn/celltalkdb/). The same intestinal cell populations were also analyzed with CellChat for systems-level analysis and better interpretation of cell–cell communication. To predict functions for poorly studied pathways, the computeNetSimilarity function was utilized for pathway analysis and netVisual_embedding function was utilized to visualize pathway clustering in 2D plot. To discover major signaling changes in response to diet, the rankNet function was utilized to compare the information flow change of each signaling pathway within the network. To predict and visualize key signaling events between cell populations, netVisual_aggregate function was utilized to visualize the signaling network of NECTIN–CD226 using immune cells and structural cells as receivers, respectively.

### Functional enrichment analysis with g:GOSt

g:GOSt analysis was performed on g:Profiler web server (https://biit.cs.ut.ee/gprofiler/gost; Raudvere et al., 2019). Common upstream regulators from HFHS diet intestine were input into the functional enrichment analysis query and then analyzed using g:GOSt functional profiling. Top differentially regulated signaling pathways were shown. Upstream regulators were considered common if implicated in 10 or more cell types from a lineage. Terms were ranked by a normalized enrichment score.

### Statistical analysis

GraphPad Prism 6 (GraphPad Software) was used for the graphic representation and statistical analysis of the data. All data were presented as the mean ± SEM. A two-tailed Student’s *t* test was used for comparison between groups. Multiple comparisons were performed using a two-way ANOVA followed by Sidak’s multiple comparisons test. P < 0.05 was considered statistically significant. ns, not significant; *P < 0.05; **P < 0.01; ***P < 0.001; ****P < 0.0001.

### Online supplemental material

Fig. S1 shows lineage-associated gene signatures of all CD45.2^+^ intestinal cells and intestine T cell clusters from the chow diet and HFHS diet intestine. Fig. S2 shows H&E staining of small intestines, gating strategy of scRNA-seq-defined CD45.2^+^ intestinal cell populations, and viability test in flow cytometry analysis. Fig. S3 shows the flow cytometry gating strategy, signature genes, viability test, and enrichment analysis of the unique T cell subsets accumulate in HFHS diet intestine. Fig. S4 shows the analysis of the macrophage populations and the comparison of key genes related to intestine barrier function and nutrient absorption function between chow diet and HFHS diet intestine. Fig. S5 shows the comparison between chow diet and HFHS diet intestine interactomes and antibody blockade experiments in vivo. Table S1 shows table of the most differentially expressed genes (DEGs) for CD8αα^+^ IEL-T cells of HFHS diet vs. chow diet. Table S2 shows table of the most DEGs for CD8αβ^+^ IEL-T cells of HFHS diet vs. chow diet. Table S3 shows the table of the most DEGs for macrophage subsets. Table S4 shows gene lists utilized to create module scores for enterocyte of villus top. Table S5 shows gene lists utilized to create module scores for enterocyte of villus bottom. Table S6 shows count of interacting ligand–receptor pairs in chow diet mice intestine interaction network. Table S7 shows the count of interacting ligand–receptor pairs in HFHS diet mice intestine interaction network. Table S8 shows table of the DEGs between HFHS diet and chow diet intestine for each cell type. Table S9 shows IPA analysis of upstream regulators of each intestine cell type. Table S10 shows the count of cell types shared by the upstream regulators of IPA analyses. Table S11 is a list of upstream regulators implicated in mouse and human studies that have been associated with immune homeostasis and obesity. Table S12 shows the top differentially regulated signaling pathways in response to HFHS diet suggested in g:GOSt functional enrichment analysis of common upstream regulators. Table S13 lists primers used for QPCR.

## Supplementary Material

Table S1 shows table of the most DEGs for CD8αα^+^ IEL-T cells of HFHS diet vs. chow diet.

Table S2 shows table of the most DEGs for CD8αβ^+^ IEL-T cells of HFHS diet vs. chow diet.

Table S3 shows table of the most DEGs for macrophage subsets.

Table S4 shows gene lists utilized to create module scores for enterocyte of villus top.

Table S5 shows gene lists utilized to create module scores for enterocyte of villus bottom.

Table S6 shows count of interacting ligand–receptor pairs in chow diet mice intestine interaction network.

Table S7 shows count of interacting ligand–receptor pairs in HFHS diet mice intestine interaction network.

Table S8 shows table of the DEGs between HFHS diet and chow diet intestine for each cell type.

Table S9 shows IPA analysis of upstream regulators of each intestine cell type.

Table S10 shows count of cell types shared the upstream regulators of IPA analyses.

Table S11 is a list of upstream regulators implicated in mouse and human studies that have been associated with immune homeostasis and obesity.

Table S12 shows top differentially regulated signaling pathways in response to HFHS diet suggested in g:GOSt functional enrichment analysis of common upstream regulators.

Table S13 lists primers used for QPCR.

SourceData F1 is the source file for Fig. 1.

SourceData FS2 is the source file for Fig. S2.

## Data Availability

All data associated with this study are presented in the article or supplemental tables. The genomics data generated during this study is available through GEO accession GSE221006. Further information and requests for new reagents generated in this study may be directed to and will be fulfilled by the lead contact, Aldons Lusis.
