# Supplementary material for: Intestinal cell type-specific communication networks underlie homeostasis and response to Western diet
Source: J Exp Med. 2023 Mar 7;220(5):e20221437. doi: 10.1084/jem.20221437 (PMC10038833; doi:10.1084/jem.20221437)
Supplement: SourceData F1 — is the source file for Fig. 1. [file JEM_20221437_SourceDataF1.pdf]

Source Data for Fig. 1F (Upper panel)

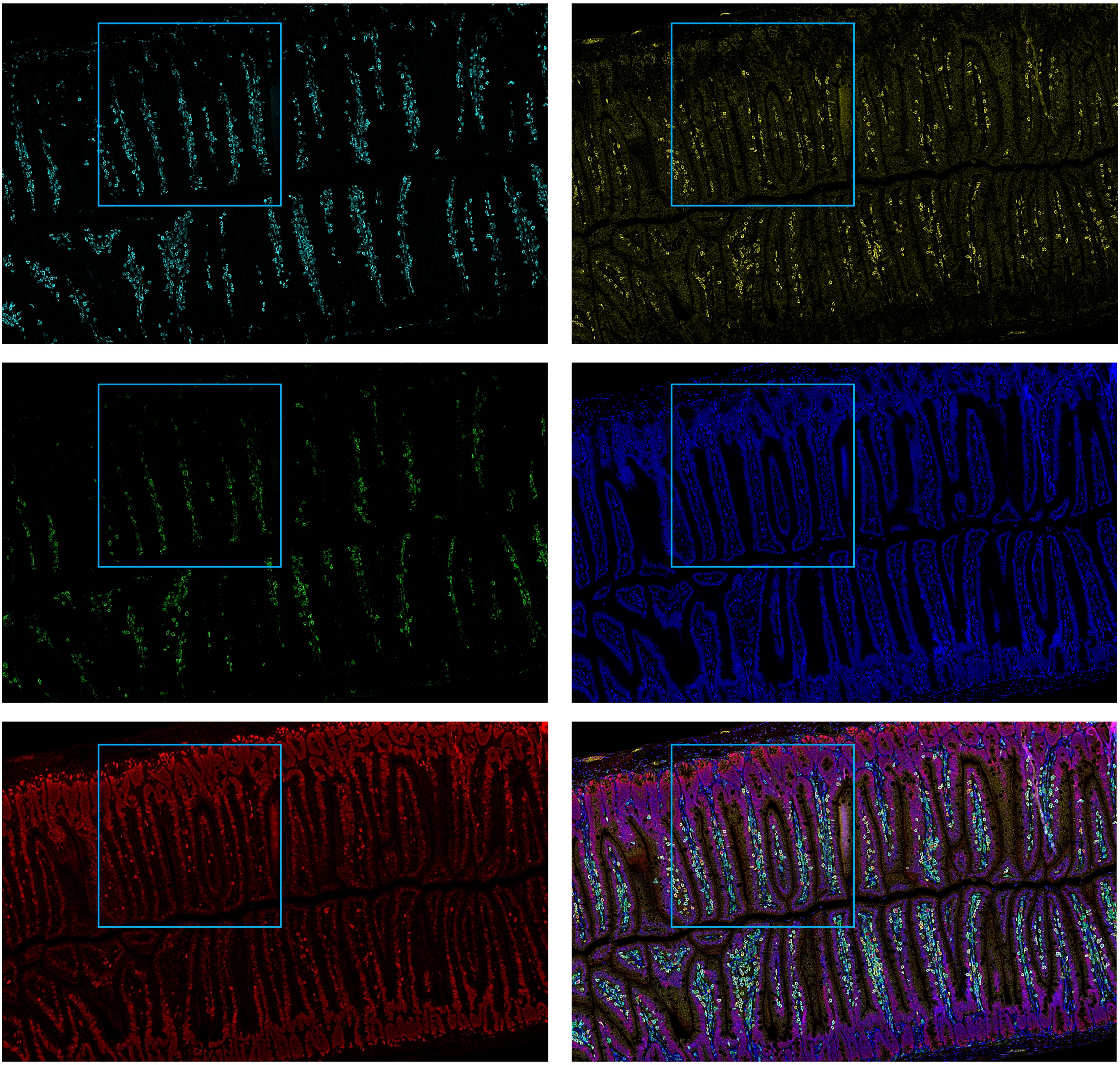

Each panel and merged image of Multiplex-Immunohistochemistry (mIHC) staining of small intestine from chow diet mice.

The blue boxes indicate the image area showed in the plots.

Source Data for Fig. 1F (Lower panel)

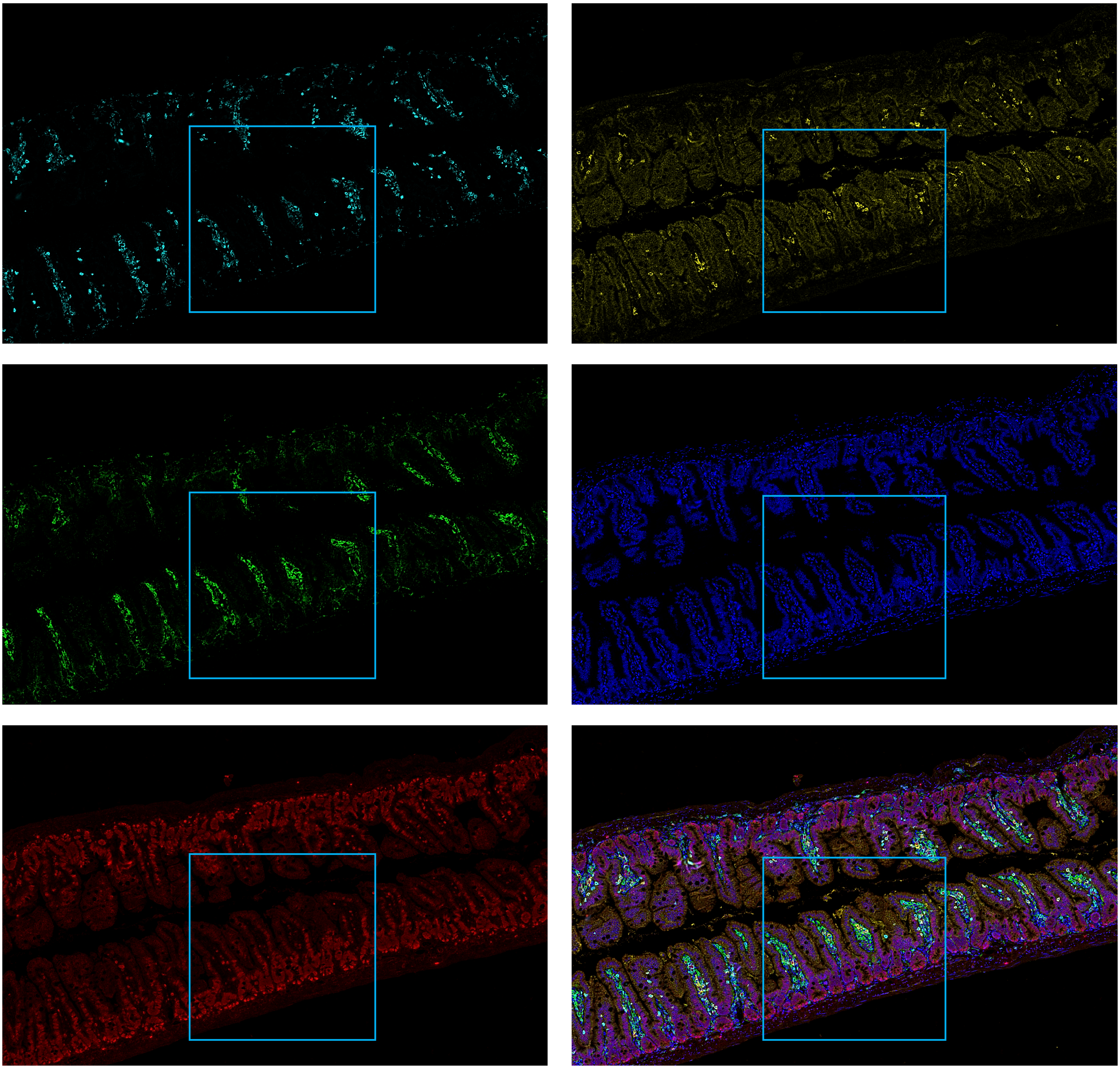

Each panel and merged image of Multiplex-Immunohistochemistry (mIHC) staining of small intestine from HFHS diet mice.

The blue boxes indicate the image area showed in the plots.
