# Supplementary material for: Intestinal cell type-specific communication networks underlie homeostasis and response to Western diet
Source: J Exp Med. 2023 Mar 7;220(5):e20221437. doi: 10.1084/jem.20221437 (PMC10038833; doi:10.1084/jem.20221437)
Supplement: SourceData FS2 — is the source file for Fig. S2. [file JEM_20221437_SourceDataFS2.pdf]

Source Data for Fig. S2A

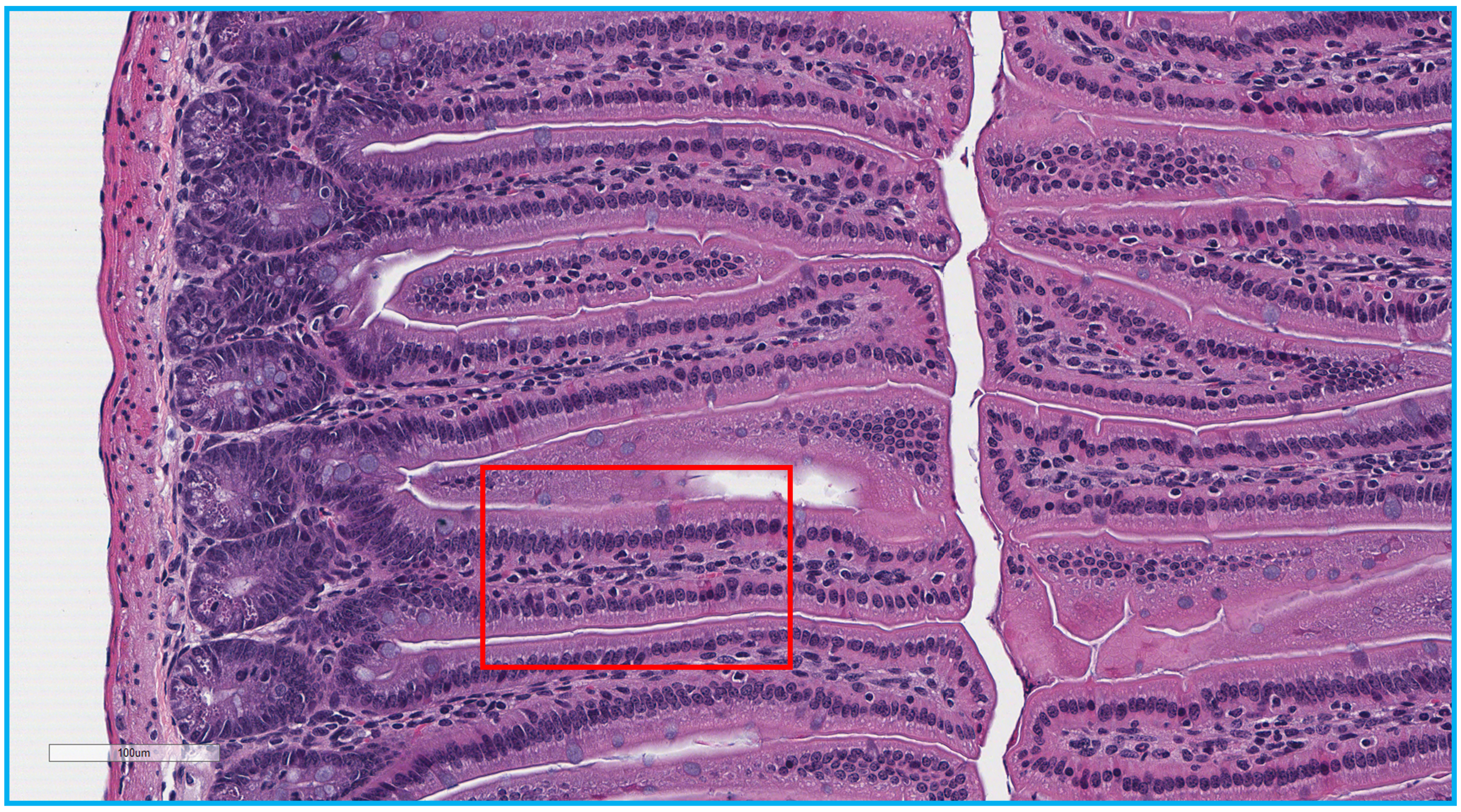

H&E staining of small intestine sections from chow diet mice.

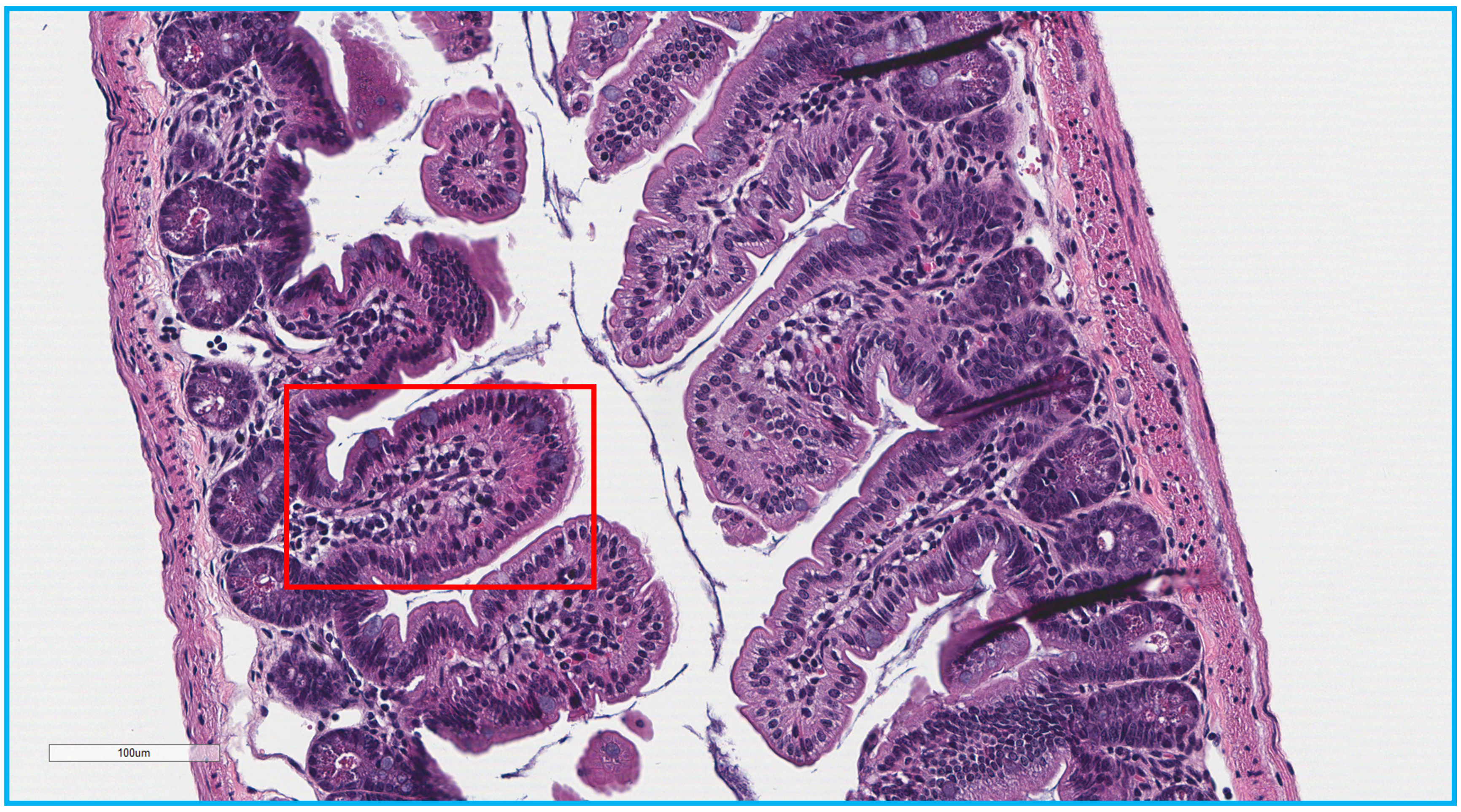

H&E staining of small intestine sections from HFHS diet mice.

The blue boxes indicate the image area showed in the plots. The red boxes indicate the magnified area.
